# The oncogene BCL6 is up-regulated in glioblastoma in response to DNA damage, and drives survival after therapy

**DOI:** 10.1371/journal.pone.0231470

**Published:** 2020-04-22

**Authors:** Marie-Sophie Fabre, Nicole M. Stanton, Tania L. Slatter, Samuel Lee, Dinindu Senanayake, Rosemary M. A. Gordon, M. Leticia Castro, Matthew R. Rowe, Ahmad Taha, Janice A. Royds, Noelyn Hung, Ari M. Melnick, Melanie J. McConnell

**Affiliations:** 1 Centre for Biodiscovery and School of Biological Sciences, Victoria University of Wellington, Wellington, New Zealand; 2 Malaghan Institute of Medical Research, Wellington, New Zealand; 3 Department of Pathology, Dunedin School of Medicine, University of Otago, Dunedin, New Zealand; 4 Neurosurgery, Southern District Health Board, Dunedin, New Zealand; 5 Weill Cornell Medical College, New York, New York, United States of America; Sechenov First Medical University, RUSSIAN FEDERATION

## Abstract

The prognosis for people with the high-grade brain tumor glioblastoma is very poor, due largely to low cell death in response to genotoxic therapy. The transcription factor BCL6, a protein that normally suppresses the DNA damage response during immune cell maturation, and a known driver of B-cell lymphoma, was shown to mediate the survival of glioblastoma cells. Expression was observed in glioblastoma tumor specimens and cell lines. When BCL6 expression or activity was reduced in these lines, increased apoptosis and a profound loss of proliferation was observed, consistent with gene expression signatures suggestive of anti-apoptotic and pro-survival signaling role for BCL6 in glioblastoma. Further, treatment with the standard therapies for glioblastoma—ionizing radiation and temozolomide—both induced BCL6 expression *in vitro*, and an *in vivo* orthotopic animal model of glioblastoma. Importantly, inhibition of BCL6 in combination with genotoxic therapies enhanced the therapeutic effect. Together these data demonstrate that BCL6 is an active transcription factor in glioblastoma, that it drives survival of cells, and that it increased with DNA damage, which increased the survival rate of therapy-treated cells. This makes BCL6 an excellent therapeutic target in glioblastoma—by increasing sensitivity to standard DNA damaging therapy, BCL6 inhibitors have real potential to improve the outcome for people with this disease.

## Introduction

The prognosis for people diagnosed with the WHO grade IV brain tumor glioblastoma is very poor, due largely to the lack of response to therapy. The gold-standard therapy for glioblastoma is surgery to debulk the tumor, followed by fractionated radiation and temozolomide chemotherapy [1]. This aims to induce significant DNA damage to the remaining, non-resected tumor—both single and double-stranded DNA breaks from radiation-induced radical species, and alkylation of purine residues by temozolomide. The expected cellular response to this DNA damage should be apoptosis. In glioblastoma, this does not occur—there is little or no apoptosis in response to therapy [2], so damaged cells continue to proliferate, exacerbating the mutagenic and genome instability effects of DNA damaging therapy. New approaches in glioblastoma such as targeted therapy and immunotherapy continue to be developed, but these have had very limited success [3]. If the block to cell death could be identified, glioblastoma could be sensitized to DNA damage induced by standard therapies, which would have an immediate impact on patient outcome.

Cell death blockade in response to DNA damage is observed during B-cell maturation, driven by the transcription factor BCL6. BCL6 dimers bind DNA using six zinc fingers at the C-terminus, and recruit co-repressors and chromatin remodeling machinery via the BTB domain to target gene loci. BCL6 is normally expressed in germinal center B-cells during class switch recombination and somatic hyper-mutation, where it represses expression of cell cycle checkpoint and apoptosis genes. This prevents the usual cellular response to double-stranded breaks, allowing cells to successfully break and rearrange immune genes to generate unique immune receptors. Due to this anti-apoptotic activity BCL6 is a strong oncogene, with ectopic expression in B-cells a key driver event in lymphoma [4, 5].

Increasingly BCL6 protein has been found in solid malignancies, including squamous cell carcinoma [6] colorectal [7] gallbladder [8], and breast cancer [9]. In most cases, BCL6 expression is associated with poor prognosis and worse outcome, although not always—BCL6 can suppress tumorigenesis in medulloblastoma [10] and is associated with a better prognosis in a subset of gastric lymphoma [11]. Occasionally the *BCL6* locus is translocated in glioma, with BCL6 expressed in association with the IDH1 mutation R132H [12]. Finally, BCL6 protein was also observed in a subset of glioblastoma, where it was correlated with increased expression of the Axl kinase [13].

Defects in the apoptotic and cell death pathways are critical in the survival of glioblastoma and resistance to treatment, and we hypothesized that these defects could be driven by BCL6 activity in glioblastoma, based on the following lines of evidence. First, apoptosis induced by chemotherapeutic agents was prevented by BCL6 over-expression in lymphoma cell lines [14]. Secondly, BCL6 was central to a receptor tyrosine kinase inhibitor (TKI) drug-resistance pathway, and BCL6 inhibition eradicated drug-resistant leukemia-initiating cells [15]. Importantly, in GBM BCL6 expression can be found associated with defects in apoptosis—for example, BCL6 and the BCL6 target gene EP300 [16] are among anti-apoptosis genes in a signature of survival in primary glioblastoma [17].

Here, we identified a series of pathways driving cell survival that are regulated by BCL6, and confirmed that BCL6 was essential for proliferation and survival of glioblastoma. Importantly, we demonstrated that DNA damaging cancer therapies up-regulated BCL6 expression *in vitro* and in an intra-cranial tumor model *in vivo*, and that blockade of BCL6 enhanced the efficacy of those therapies.

## Materials and methods

### Tumor immunohistochemistry

Sixty-two glioblastomas were obtained from the neurosurgical unit in Dunedin, New Zealand, predominantly primary glioblastoma but with a low frequency of secondary disease. The study had national ethics approval from the Multi-region Ethics Committee (MEC/08/02/016/AM01) and all patients provided written informed consent at the time of surgery. DNA was extracted from each tumor and the p53 gene sequenced to identify tumors with either wild-type or mutant TP53. Exon 4 of both the IDH1 and IDH2 genes were also sequenced to identify oncogenic mutations [18]. Tissue sections from paraffin-embedded material were subjected to heat-mediated antigen retrieval. BCL6 staining used the PG-B6p primary antibody (Dako, Glostrup, Denmark). MGMT staining used the MT3.1 primary antibody (Abcam, Cambridge, UK). Positive cells were identified using EDL (Dako) and DAB. Positive cells were counted in at least 10-high-powered fields (x400 magnification) using the Aperio Scancope CS digital pathology system (Aperio, Vista, CA, USA) and the percentage of positive cells per total cells measured. MGMT positive tumors had >30% positive tumor cells.

### Cell lines

Human GBM lines LN18, U87-MG and T98G were obtained from ATCC and used within 20 passages. NZG-1003 and NZG-0906 primary GBM cell lines were previously derived from primary tumors in our laboratory [18, 19]. Raji cells were a gift from Ian Morison, University of Otago, New Zealand. All cells were maintained at 37°C/5% CO2 and cultured in RPMI-1640 with 10% FBS without antibiotics. Regular PCR testing (e-Myco, Intron BioTechnology, Korea) showed all cultures were mycoplasma free. No further authentication was performed.

### Intra-cranial tumors

All experiments using mice were conducted in accordance with the New Zealand Animal Welfare Act 1999 (www.legislation.govt.nz/act/public/1999/0142/latest/DLM49664.html*)* and were approved by the Victoria University of Wellington Animal Ethics committee, approval 2012R7M. C57BL/6J mice were originally obtained from Jackson Labs (Bar Harbour, ME) and bred at the Biomedical Research Unit of the Malaghan Institute of Medical Research. Animals were housed in small groups in specific pathogen free conditions at 22°C with a 12 hr light/dark cycle and *ad libitum* access to food and water. Environmental enrichment items such as nesting wool (PuraWool, Able Scientific, Australia) and cardboard tubes were provided at all times. Six male mice, 8 weeks old, weighing 25–30 g, were injected intra-cranially with 25,000 viable GL261 cells. Animals were anaesthetized by intra-peritoneal injection of xylazine (100 mg/kg) and ketamine (10 mg/kg), (Phoenix Pharm.), then Lacri-Lube (Allergan) was applied across the cornea of the eye and animals kept warm on a heat pad. A burr hole was drilled in the skull 0.1 mm posterior to the bregma and 2.3 mm lateral to the midline. Cells in 2 μl of PBS were administered stereotactically (Stoelting Apparatus), into the burr hole, using a Hamilton syringe with a 32-gauge needle. The needle was advanced to a depth of 2.3 mm from the brain surface and the cell suspension delivered slowly over the course of 2 to 3 minutes. Following injection, the needle was left in place for 2 minutes, after which time, it was raised to a depth of 1.5 mm below the brain surface and left in place for an additional minute. On withdrawal of the needle, the burr hole was sealed with bone wax and the incision sutured. Animals were monitored daily, and received sub-cutaneous analgesics (Carprofen (5 mg/kg), Norbrook Laboratories, and Buprenorphine (0.1 mg/kg), Renckitt Benckiser Pharmaceuticals), to control post-operative pain. Animals were randomly assigned into control and treatment groups (n = 3/group). At the onset of symptoms (at least two consecutive days of weight loss), the treatment group were exposed to 10 Gy of whole-brain ionizing radiation [20]. They were culled by CO_2_ inhalation either 24 or 48 hours after treatment, then cardiac perfusion with saline performed before cervical dislocation and collection of brain tissue. The non-irradiated tumor bearing mice were culled at the ethical endpoint of 10% weight loss or onset of neurological symptoms, according to the animal ethics protocol. Brains were embedded in OCT (Tissue-Tek), frozen and kept at -80°C until sectioning.

### Drugs

Doxorubicin (Merck, Billercia, USA) was dissolved at 10mM in PBS. Cells were treated with 1–3 μM and harvested at 1–3 days. TMZ (methazolastone, SelleckChem, Houston TX, USA) was dissolved in PBS at 0.33 mg/mL, and cells treated with 10 μM TMZ every 2 days for 7 treatments. Cells were harvested 2 days after the last treatment. Cells received ionizing radiation from a cesium-137 source (Gammacell 3000 Elan, Theratronics, Ottawa, CA). After irradiation, cells were incubated for 24 hours before harvest. The peptide mimetic inhibitor RI-BPI [21] was dissolved in sterile pre-gassed water pH6.2, at 50°C and used at 2 μM. The small molecule inhibitor FX1[22] was dissolved in DMSO at 25 mM.

### Antibodies

Mouse monoclonal anti-Bcl-6 D8, anti-mouse IgG-HRP and anti-rabbit IgG-HRP were from Santa Cruz Biotechnology, (Santa Cruz, CA); mouse monoclonal anti-α-Tubulin Clone B-5-1-2 was from Sigma-Aldrich (Auckland, NZ), goat anti-mouse Alexa 488 from Invitrogen (ThermoFisher Scientific, Auckland, NZ); FITC anti-active Caspase3 and annexinV-APC (BD Pharmingen, Auckland, NZ); mouse monoclonal anti-BCOR, mouse monoclonal anti-NCOR2 (SMRT), and mouse monoclonal anti-β-actin were from Sigma-Aldrich (Auckland, NZ); goat polyclonal anti-BCOR and rabbit polyclonal anti-NCOR were from Abcam (Cambridge, UK); mouse monoclonal anti-SMRT was from GeneTex (Irvine, CA.)

### Western blotting

Soluble protein was extracted into lysis buffer (140 mM NaCl, 50 mM Tris pH 7.5–8, 1% triton, protease inhibitor (Complete EDTA free, Roche, Auckland NZ)) and quantified using the DC assay (Bio-Rad, Auckland NZ). Forty micrograms of protein was electrophoresed through 10% SDS PAGE and transferred to PVDF membrane (Bio-Rad, Auckland, NZ). After blocking in 3% skim milk at room temperature, the upper part of the membrane was incubated with 1:500 (3% skim milk) of anti-BCl6 monoclonal antibody D8 and lower part with 1:2,000 (3% skim milk) of anti-tubulin. Goat anti-mouse IgG HRP secondary antibody was used at 1:10,000 (3% skim milk). Detection by enhanced chemiluminescence (Ultrasignal ECL kit, Pierce) was imaged with Gel Logic 4000 PRO Imaging System (Carestream, Rochester, NY USA). Raji lysate was a positive control for BCL6 expression. Co-repressor blots were done as above, but whole cell lysate electrophoresed through 6% acrylamide gels, and blocked in 5% BSA. All co-repressor primary antibodies were used at 1:1000 dilution. ECL detection used Western Lightning Pro (Perkin Elmer, Waltham MA), imaged on the Amersham Imager 600 (GE Healthcare Life Sciences, Auckland, NZ).

### Immunofluorescence

Cells grown on sterile glass coverslips were fixed 15 minutes in 4% paraformaldehyde, then permeabilized 15 minutes on ice in 0.1% Triton X-100 in PBS. Samples were blocked in 3% BSA in PBS 1 hour then anti-BCL6 antibody (1:50) was added and incubated 4°C overnight. Coverslips were washed in PBS then incubated 1:500 anti-mouse Alexa 488 secondary antibody, 3% BSA in PBS, room temperature 1 hour. After washing, samples were mounted by inversion onto mounting medium on glass slides. Images were acquired using a BX51 compound microscope (Olympus, Auckland NZ). For analysis of tumors, slides were thawed at room temperature for 10 minutes then rehydrated in PBS for 10 minutes. Non-specific binding was prevented by blocking antigenic sites for 30 minutes in PBS containing 1% FBS. A mouse monoclonal anti- BCL-6 antibody (Dako PG B6p at 1/20 or D8, at 1/50) was diluted in incubation buffer (PBS containing 1%FBS and 3% triton X-100) and applied overnight at 4°C. Slides were then washed 3x 15 minutes in PBS. A goat anti mouse IgG labelled with AF488 (A-11029, 1/250 dilution) was used as a secondary antibody and incubated for 1 hour at room temperature. After 3 washes in PBS, slides were mounted using ProLong^™^ Gold Antifade Mount (ThermoFisher Scientific, Auckland, NZ).

### Transfection

pcDNA3-hBCL6-DZF-GFP, pcDNA3-GFP, BCL6-4xtkLUC, and pGL3 control plasmids were purified using PureLink^®^ HiPure Plasmid Filter Maxiprep Kit (Invitrogen, Auckland NZ). All cells were transfected using Viafect (Promega, Auckland NZ) at 70% confluence following manufacturers instructions, and cells were harvested at 24–48 hrs. siRNA against BCL6 and control (Santa Cruz Biotechnology, Santa Cruz, CA, USA) were used according to the manufacturers siRNA gene silencing protocol. For hypoxic transfection, cells were pre-incubated in 0.5% oxygen for 24 hours, then transfected and replaced into 0.5% oxygen for an additional 24 hours.

### ZFN genome editing

LN18 or U87-MG at approximately 70% confluence were transfected with each of PZFN1 and PZFN2 CompoZr^™^ Knockout Zinc Finger Nuclease plasmids targeted to BCL6 (Sigma Aldrich, USA), using Lipofectamine^®^ 3000. After both 24 hours and 72 hours, the medium was collected and the debris and detached cell fraction was pelleted by centrifugation, and the supernatant aspirated. The adherent cells were then harvested and pelleted by centrifugation, or combined with the non-adherent fraction before pelleting by centrifugation. Cell pellets were stored at -80° C. gDNA was extracted from ZFN transfected cell pellets using the Quick-gDNA^™^ MiniPrep kit (Zymo Research, USA) as per the manufacturers protocol. gDNA was quantified by fluorometry before PCR amplification of a 381 bp fragment of the BCL6 using Phusion^®^ High-Fidelity DNA Polymerase, and BCL6 locus forward primer (5’-GAAGAGTTCCTCAACAGCCG-3’), and reverse primer (5’-TTCTGGGATTGTTTCCTTGG-3’), using the following parameters: 1 cycle of 98 °C, 30 s. 30 cycles of 98 °C, 10s; 57 °C, 30 s; 72°C, 10 s. Nuclease activity was detected by CEL1 endonuclease digestion of heteroduplex DNA using the Surveyor^®^ Mutation Detection Kit (IDT, USA) using the manufacturers protocol.

### Clonogenic assay

Transfected cells were flow sorted according to GFP status (Influx, BD Biosciences) then 400, 200 and 100 GFP positive or negative cells were seeded into 10 cm dishes and incubated for 2 weeks. Colonies were washed twice with PBS followed by fixation with neutral buffered formalin, 6% v/v for 30 minutes followed by overnight staining with 0.5% methylene blue, on a rocking platform. Plates were washed in cold tap water until the water ran clear, drained and allowed to dry. Plates were scanned (GE ImageScanner III) and colonies >50 cells were counted using Fiji (ImageJ2) software. Plating efficiency and surviving fractions were calculated as follows: Plating efficiency (PE) = number of colonies counted / number of colonies plated. Surviving fraction (SF) = PE sample / PE control.

### Cell death and apoptosis

Cells were washed in PBS+1% BSA with 50 ng/mL PI, and PI positive cells were identified by standard flow cytometry techniques using the LSRII flow cytometer with FACSDiva 6.2 acquisition software (BD Biosciences, Auckland, NZ). Data were analyzed using FlowJo software (FlowJo, Ashland, OR, USA). Annexin V positive cells were detected using either AnxV-FITC or AnxV-APC (ThermoFisher Scientific, Auckland NZ). For Caspase3 activation, cells were trypsinized, washed in PBS then fixed and permeabilized using the FoxP3 Fixation/Permeabilization Concentrate and Diluent Reagent (e-Biosciences, San Diego, CA). Fixed-permeabilized cells were stained overnight with FITC anti-Active Caspase3 apoptosis kit (BD Pharmingen, Auckland NZ) then washed and analyzed.

### Luciferase reporter assay

Luciferase activity was assessed with the Promega Luciferase Assay System (Madison, WI). Cells were imaged to determine density, then washed in PBS and then lysed and scraped in cell culture lysis reagent (25 mM Tris-phosphate, 2 mM Dithiothreitol, 2 mM 1,2-diaminocyclohexane-N,N,N’,N,-tetraacetic acid, 10% (v/v) glycerol, 1% (v/v) Triton X-100). Lysate was added to a 96 well solid white flat bottom plate (Corning, NY). Luciferase assay reagent (LAR) was prepared according to the manufacturer’s instructions. LAR was injected into the plate and read by the Tecan Infinite M1000 Pro Plate Reader (Männedorf, Switzerland). Light values were normalized to cell counts (luciferase value/number of cells) taken before lysis of the cells and then relative change (double transfection or treated value/single transfected untreated value) value in light compared to untreated single-transfected control was calculated.

### Quantitative reverse transcriptase PCR (q-RT-PCR)

RNA was extracted using the Zymo Quick RNA MiniPrep kit (Zymo Research, Irvine, CA) according to the manufacturer’s instructions. RNA was quantified with the Qubit RNA high sensitivity assay (Life Technologies, Auckland, NZ) according to the manufacturer’s instructions. Reverse transcription was performed using the iScript cDNA synthesis kit (BioRad, Auckland, NZ) according to the manufacturer’s instructions, with 250 ng of RNA in each reaction. qPCR was performed with KAPA SYBR^®^ FAST Universal One-Step qRT-PCR Kit (KAPA Biosystems, Wilmington, MA) and Qiagen Quantitect Primer assays (Qiagen, Hilden, Germany) for BCL6, BCOR, HPRT, NCOR1, and NCOR2 (SMRT) with 4 μl cDNA per reaction. ΔCt was calculated relative to HPRT.

### RNA-sequencing and gene set enrichment analysis

RNA was extracted as above, then polyA+ RNA TruSeq libraries generated and 2x 125 bp paired end sequenced, using one lane of an Illumina HiSeq. Paired-end RNA-seq fastq files were processed using Trimmomatic (v0.36) to remove Illumina adaptors and low-quality sequences, then read quality assessed using FASTQC (v0.11.4). Transcript abundances were quantified using Kallisto (v0.43.1) by pseudo-alignment to the GRCh38 Human cDNA transcriptome (release 89) and collapsed to the gene level using tximport (v1.4.0). The R package DESeq2 (v1.16.1) was used to test for differential gene expression between experimental conditions using the Wald test. Log2 fold-changes were moderated to reduce dependence of differential expression on mean expression for genes. Genes were considered to be differentially expressed if they had a Benjamini-Hochberg adjusted p-value < 0.05 and a |log2FC |> 1.5. Code for execution used our own pipeline (https://www.github.com/samleenz/rnaseq_pipe). GSEA was conducted using GSEA (v3.0) run in Pre-ranked mode against the MSigDB (v6.1) Hallmarks collection. To create the ranked gene list differential expression results were first filtered for adjusted p-value < 0.05 and a |log2FC |> 0.5. The filtered list was ranked using the formula (score = sign(log2FC) × -log10(adjusted p—value)). GSEA was then run using 1000 permutations and a classic scoring scheme, with results filtered for significance using a FDR adjusted p-value < 0.25.

### Migration scratch assay

Cells were seeded into 24 well plates at 0.75 x 10^5^ cells per well and left to adhere overnight and form a monolayer before scratching was made using a 200 μl pipette tip using an apparatus to ensure reproducible scratches in the monolayer. Cells were then rinsed twice with PBS and drug added in RPMI with reduced 0.1% FCS to ensure migration, rather than division, was assessed. Scratches were imaged using a phase-contrast inverted microscope (Olympus IXS1, Olympus, Tokyo, Japan), before being incubated for 17 hours at 37°C, then imaged again. Images were analyzed using the FIJI MRI wound healing macro tool (http://dev.mri.cnrs.fr/), and the area gained was calculated by subtracting the area of the scratch at t17 from the area of the scratch at t0.

## Results

### BCL6 regulated expression of pro-survival genes and pathways

**Fig 1:**
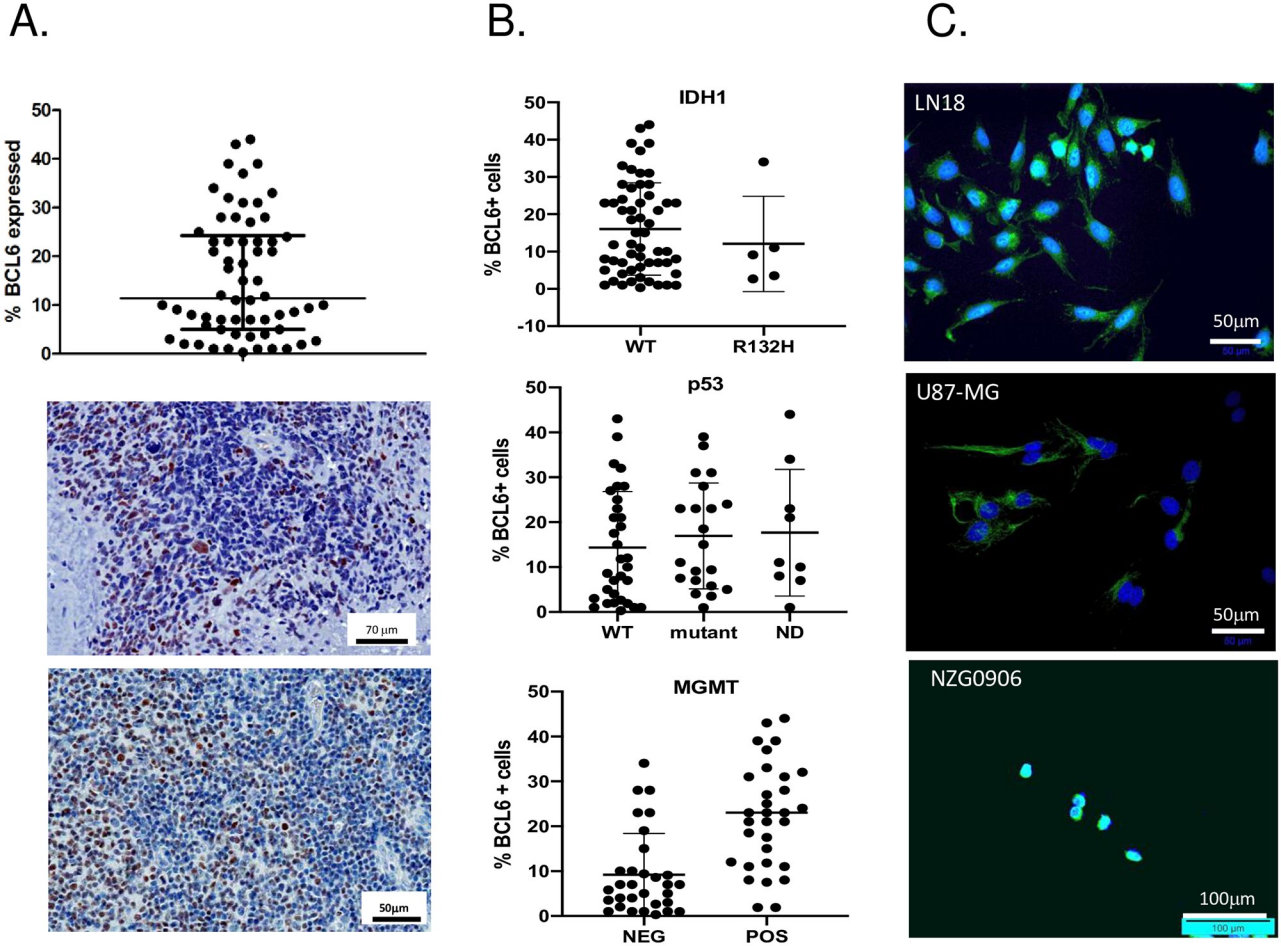
BCL6 is expressed in glioblastoma tumors and cell lines. **A**. Proportion of BCL6+ cells in 62 human GBM tumors (top), and representative IHC staining of 2 of 62 human GBM tumors, highlighting heterogeneous BCL6 protein (brown staining). **B**. BCL6+ GBM tumors after stratification by IDH1 mutation (top) p53 mutation (middle) and MGMT expression (bottom). **C**. Immunofluorescent staining for BCL6 in glioblastoma cell lines LN18, U87-MG and NZG-0906. Immunofluorescence data are representative of at least 3 independent experiments. Adjustments to brightness and contrast were used to improve visibility of fluorescent signal, and were applied to the whole image and to each image equally.

BCL6 protein expression was examined by immunohistochemistry in 62 GBM tumor specimens (Fig 1A). BCL6+ cells could be found in every tumor section examined, from 0.96–45% positive tumor cells, with an average of 10% of cells within a tumor expressing BCL6 protein (Fig 1A). In many sections the BCL6+ cells appeared to be perivascular, so the variation in the number of BCL6+ tumour cells between samples may represent a coincidental difference in the vascularity of sections sampled. Given that BCL6 is expressed in hematopoietic cells, we examined the morphology of the BCL6 positive cells, but did not observe macrophages or lymphocytes expressing BCL6. Expression was only on the tumor cells. Next, the IDH1 and IDH2, MGMT and p53 status of each tumor was determined and correlated to BCL6 expression (Fig 1B). There were no tumors with IDH2 mutations, and only 5/62 with IDH1 R132H mutations. These had the same range of BCL6+ cells as the IDH1 wild-type cells. Similarly, the proportion of each tumor that was BCL6 positive was similar regardless of whether the tumor had wild-type or mutant p53. Intriguingly, MGMT expressing tumors had a higher proportion of BCL6-positive cells than the MGMT-negative tumours.

We compared the expression and subcellular localisation of BCL6 in a panel of glioblastoma cell lines, by immunofluorescence microscopy (Fig 1C). BCL6 protein level varied—the LN18 cell line had substantial nuclear and cytoplasmic expression in most cells, as demonstrated by the cyan (green+blue) staining of the nuclei. U87-MG had cytoplasmic BCL6 in all cells and the primary tumor-derived line NZG-0906 had strong nuclear expression in the majority of cells. NZG-0804, another primary glioblastoma line [19, 20], had high levels of nuclear BCL6 in about half of all cells, with cytoplasmic BCL6 also present, while the primary line NZG-1003 had no basal expression (S1 Fig).

**Fig 2:**
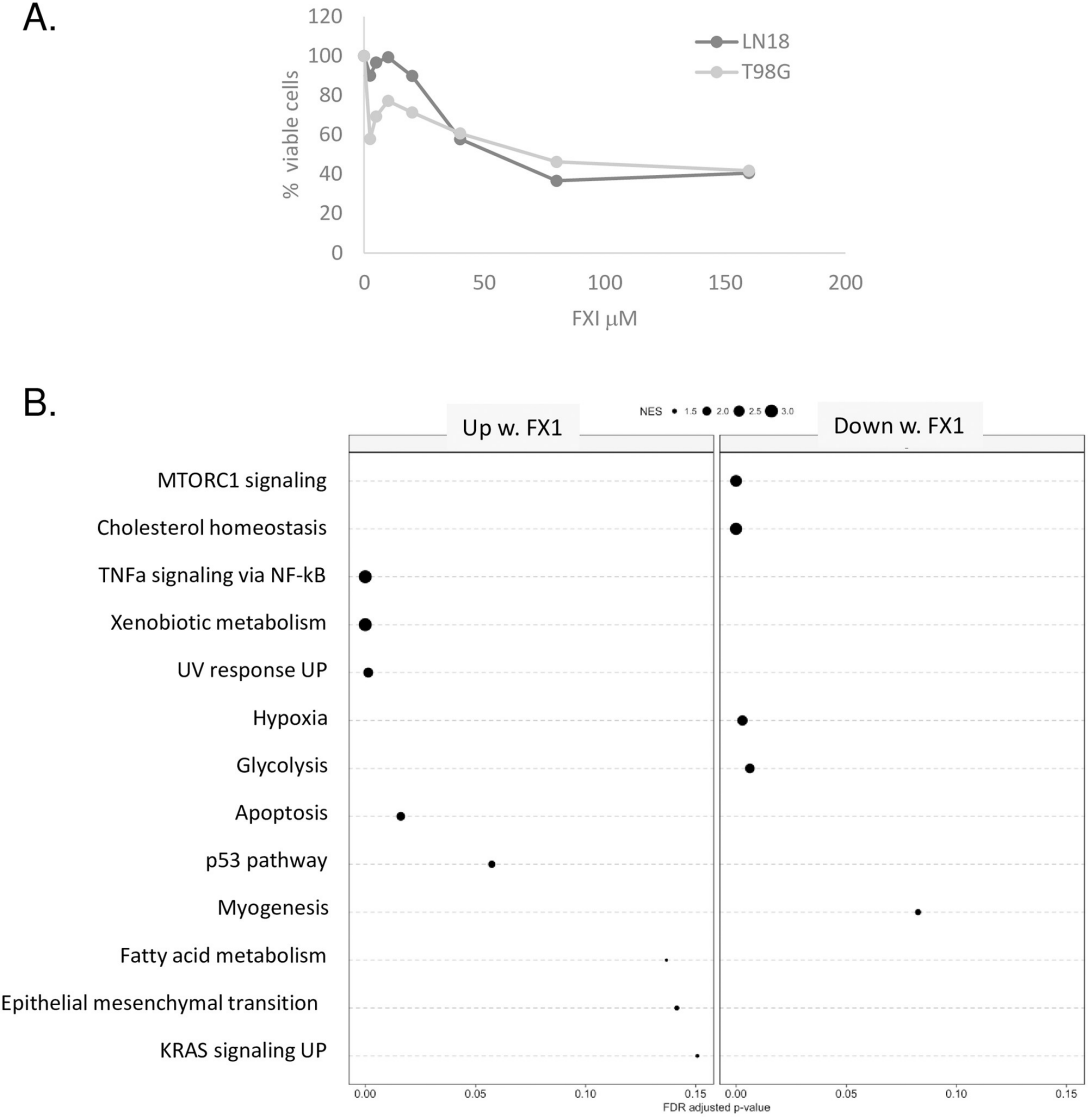
The BCL6 inhibitor FX1 alters expression of multiple hallmarks of cancer in glioblastoma. **A**. LN18 and T98G cells were treated with a range of FX1 concentrations for 48 hours, then metabolic activity measured using reduction of the formazan salt MTS. Data are the average of triplicate experiments. **B**. In triplicate, LN18 cells were treated with 25 ⌠M FX1 or the corresponding dose of DMSO for 24 hours, then RNA extracted and polyA+ RNA sequenced. Genes that were differentially expressed in FX1 versus DMSO samples were separated into up-regulated and down-regulated, then a gene set enrichment analysis was performed for the ‘hallmarks of cancer’ gene sets. The size of the dot represents the enrichment score for each gene set, and the position along the x-axis indicates the level of statistical significance attained.

Prior reports suggested that BCL6 expression was correlated to apoptosis resistance [12] or induction of senescence [13]. To confirm this, and to determine what other cellular pathways were regulated by BCL6 in glioblastoma, a panel of immortalized glioblastoma cell lines were treated with the small molecule BCL6 inhibitor FX1, which blocks co-repressor interaction with the lateral groove of the BTB domain [21]. For the majority of cell lines tested, the IC50 was approximately 40 μM (Fig 2A), similar to that of lymphoma cell lines [21]. RNA from cells treated with 40 μM FX1 was of poor quality, reflecting the reduced cellular viability, so RNA was extracted from the LN18 cell line 24 hours after treatment with either 25 μM FX1, or DMSO as a vehicle control, then RNA-sequencing of poly-adenylated transcripts performed. Differentially expressed transcripts were identified, and divided into ‘up-regulated’ and ‘down-regulated’ in response to BCL6 inhibition. These were then analyzed by gene set enrichment analysis, using the ‘hallmarks of cancer’ gene set [22]. All gene sets with significant enrichment are shown (Fig 2B). The size of the enrichment score is reflected in the size of the dot, and the extent of statistical significance is shown by position along the X axis. Consistent with prior reports [12, 13], the apoptosis pathway was significantly up-regulated, confirming that BCL6 does indeed have anti-apoptotic activity in glioblastoma. Importantly, after BCL6 inhibition, the TNFα/NFκB hallmark was significantly up-regulated, consistent with the known role for BCL6 in down-regulation of the NF-κB pathway [23, 24]. In addition, upregulation of xenobiotic metabolism genes was observed, as per exposure to the xenobiotic FX1. Alterations in the hypoxia and glycolysis pathways were significantly down-regulated by BCL6 inhibition. Other hallmarks had lower enrichment scores.

### BCL6 blocks apoptosis in glioblastoma

**Fig 3:**
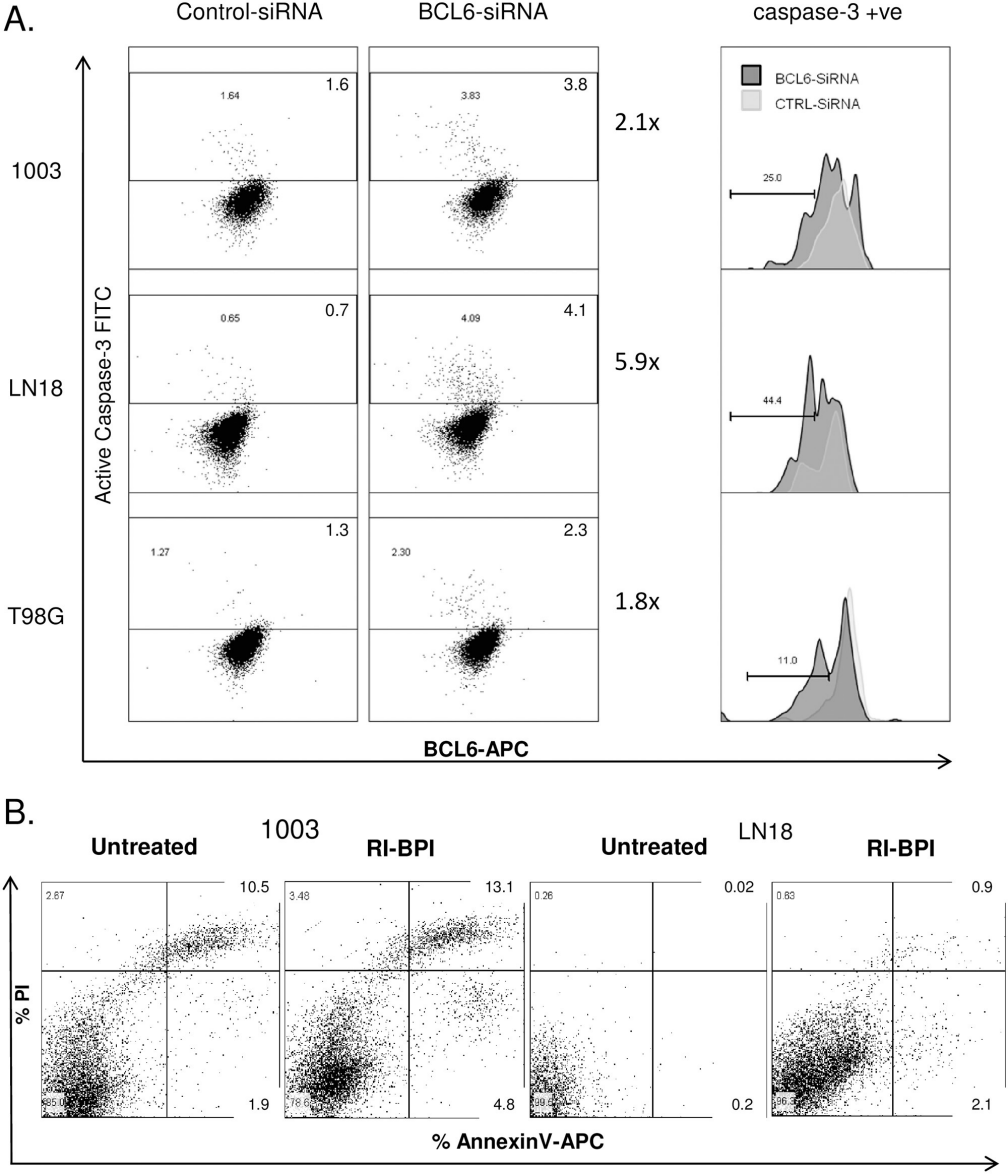
Both reduction in BCL6 level and blockade of the lateral groove increased apoptosis. **A**. NZG-1003, LN18 and T98G cells were transfected with control (left panel) or BCL6 (center panel) siRNA, and cells with active Caspase 3 determined by flow cytometric analysis (y-axis). Caspase-3 positive apoptotic cell gate was established from cells stained with a control antibody, and the percentage of cells in the apoptotic gate is given. BCL6 level was measured in parallel by intracellular staining (x-axis). In the center panel, BCL6 level was determined in the apoptotic population, and the control siRNA (light grey) compared to the BCL6 siRNA (dark grey). The median fluorescence intensity for each population is shown, data are representative of at least 3 independent experiments. **B**. NZG-1003 and LN18 cells were treated with the BCL6 peptide mimetic inhibitor RI-BPI, or a vehicle control, for 24 hours and annexin V/PI staining used to identify apoptotic and non-viable cells. Proportion of cells is given for each quadrant. All data are representative of multiple independent experiments.

There was a marked up-regulation of genes expressed in apoptosis with BCL6 inhibition, in line with BCL6 anti-apoptotic activity [15, 25–27]. To demonstrate a functional increase in apoptosis with BCL6 reduction in glioblastoma, multiple approaches were taken to block BCL6 activity. Firstly, BCL6 transcript was reduced by siRNA in three different lines—LN18, NZG-1003, and T98G, and the effect on apoptosis quantified by analysis of active caspase-3 (Fig 3A). Consistent with reported poor apoptosis in glioblastoma, the total number of cells with active caspase-3 was low, but reproducibly increased from control- to BCL6-siRNA treated cells, 2-fold for NZG-1003 and T98G, and 7-fold in LN18. This low level of induction was likely in part related to low efficacy of siRNA knockdown, and heterogeneity in BCL6 expression in the cell lines. However, specific interrogation of just the caspase-positive cell population showed a second population of apoptotic cells emerged, with less BCL6 (Fig 3A, right panel), as expected for a protein with anti-apoptotic action.

In parallel, BCL6 was inhibited using the peptide mimetic inhibitor RI-BPI, which specifically blocks BCL6 co-repressor interaction by binding in the lateral groove of the BTB domain, a major protein interaction domain [28]. Both NZG-1003 and LN18 cell lines were treated with 2 μM RI-BPI for 24 hours, then apoptosis quantified by annexin-V positivity. Consistent with the caspase-3 data, an annexin-V positive apoptotic phenotype emerged with BCL6 inhibition (Fig 3B). Again, only a small population of cells were involved. In LN18 cells, AnxV+/PI- cells increased 10-fold, from 0.2% to 2.1%, while the AnxV+/PI+ increased from 0.02% to 0.9%. This led to a 10-fold increase in the total AnxV+ apoptotic cells, from 0.2% to 3%. In NZG-1003, total AnxV+ cells increased by 50% with BCL6 inhibition, from 12.4 to 17.9%. There was a 2.5-fold increase in AnxV+/PI- cells, from 1.9% to 4.8%, as well as increased anxV+/PI+ population. Again, the cell numbers were low but the data were reproducible across 3–5 independent experiments. Interestingly, the effect of total BCL6 reduction (siRNA) was equivalent to specifically blocking protein-protein interaction at the BTB pocket (RI-BPI), suggesting that the BTB pocket may be a key mediator of BCL6 activity in glioblastoma.

### BCL6 was required for survival and long-term proliferation in glioblastoma

**Fig 4:**
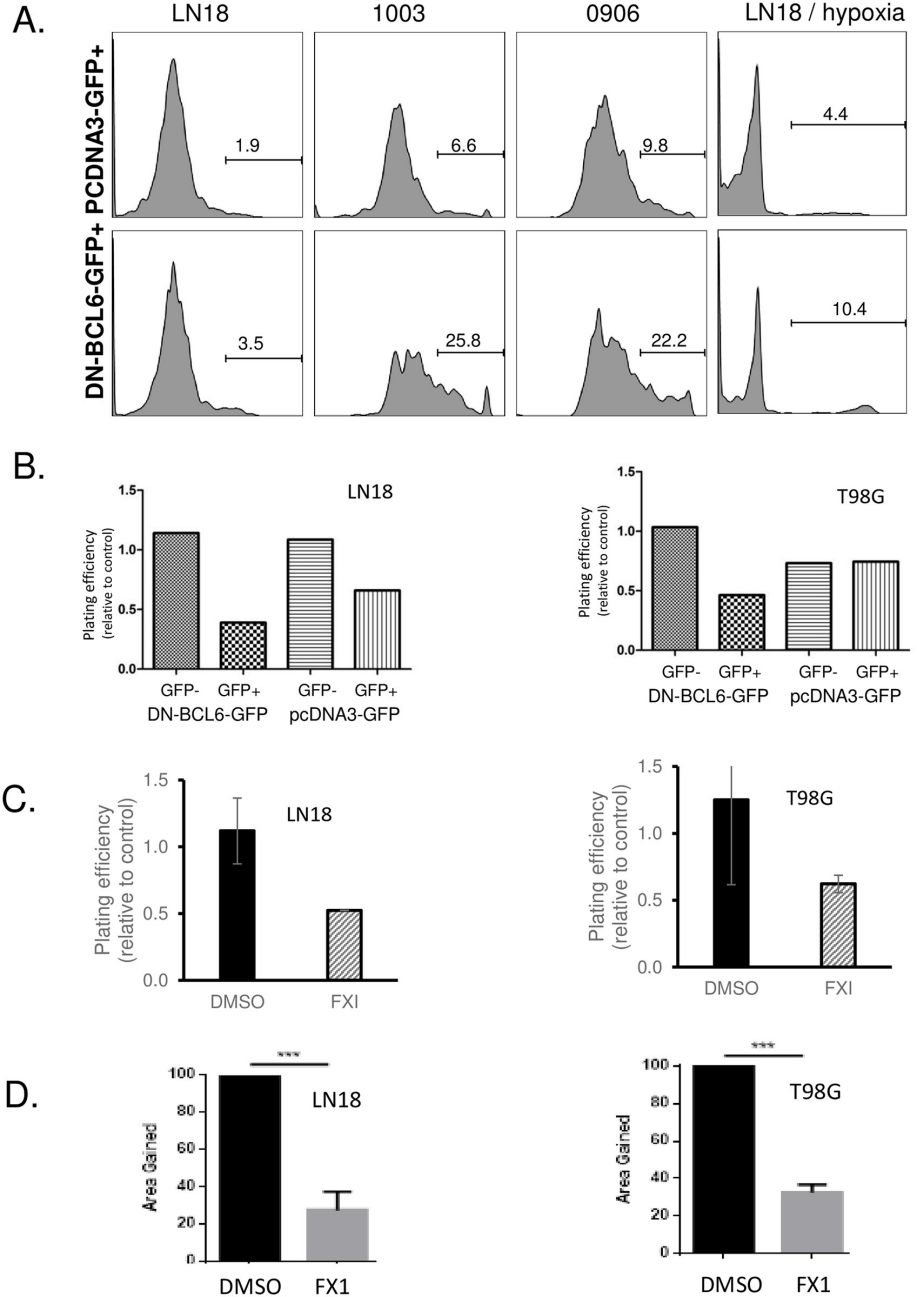
Both BCL6 inhibition by dominant negative BCL6 and blockade of lateral groove decreased viability and clonogenic potential. **A**. LN18, NZG-1003 and NZG-0906 were transfected with either a control GFP construct (upper panels) or a DN-BCL6-GFP construct (lower panels). After 24 hours, GFP positive cells were gated and propidium iodide positivity analyzed by flow cytometry as a measure of non-viability. Histogram gates were established on unstained cells, and the number of cells given for each gate. Hypoxia—LN18 cells were grown in 0.5% oxygen for 24 hours, transfected with either GFP or DN-BCL6-GFP, and replaced into 0.5% oxygen for 24 hours before analysis of GFP+ PI+ cells. **B**. GFP+ and GFP- cells were physically sorted from LN18 and T98G cells transfected with GFP, or DN-BCL6-GFP, then clonogenic plating efficiency determined for GFP+ and GFP- cells from each transfection. **C**. LN18 and T98G cells were treated with 25 μM FX1 for 24 hours, then clonogenic plating efficiency determined. All data shown are representative of at least 3 independent experiments. **D**. Migration was measured by scratch wound healing assay, data are normalized to the vehicle-treated cells, and the average +/- SEM from replicate experiments.

Despite the reported association between BCL6 and apoptosis in glioblastoma, and the significant increase in apoptosis gene set expression, none of the inhibitory approaches induced widespread apoptosis. However, the induction of apoptosis *per se* is not critical for effective therapy—as long as cells die, the actual mechanism is arguably unimportant. To look at “all death” acutely after BCL6 blockade, we examined uptake of the viability dye propidium iodide. Three cell lines were transfected with DZF-BCL6 -GFP, a BCL6 construct with intact BTB dimerisation domain, but a deletion in the zinc finger domain. This dominant negative protein dimerizes with and inhibits endogenous BCL6 binding DNA, hence blocking transcription activity of the endogenous protein [29]. GFP expression was used to identify and purify transfected cells. The GFP+ cells were gated and the effect of DZF-BCL6-GFP on viability determined as the proportion of PI+ GFP+ cells (Fig 4A), compared to the effect of GFP alone. GFP expression had a negligible effect in LN18 cells, but was detrimental to NZG-0906 and NZG-1003, with 9 and 16% of GFP+ cells PI+ after 24hrs. In LN18 and NZG-0906, DZF-BCL6-GFP expression doubled the proportion of PI+ cells, from 2–4% and from 16–31% respectively. In NZG-1003, DZF-BCL6-GFP caused a 4-fold increase in non-viable cells, from 9–36%. This activity was conserved in hypoxic conditions—hypoxia alone increased PI+ cells, but inhibition of BCL6 by DN-BCL6-GFP still increased the number of non-viable cells 2.5 times, from 4% to 10% (Fig 4A, right panel).

The proportion of cells that were non-viable immediately after BCL6 inhibition was reproducible, but small. To determine the long-term consequence of BCL6 inhibition, transfected DZF-BCL6-GFP+ cells were sorted from the non-transfected GFP- cells, and the clonogenic potential of each sub-population compared (Fig 4B), again with GFP+ cells as a control. Relative plating efficiency, the number of colonies formed from a specific number of sorted GFP+ cells, was determined by comparison to colonies from the same number of sorted GFP- cells, and was determined for DZF-BCL6-GFP and GFP alone. GFP alone had a detrimental effect on LN18 cells, with a 30% reduction in plating efficiency. However, DZF-BCL6-GFP doubled the effect, with a 60% reduction. In T98G there was no effect of GFP expression on long-term survival, and greater than 50% reduction in plating efficiency with DZF-BCL6-GFP, demonstrating clearly that loss of BCL6 activity was sufficient to reduce long term proliferative potential.

Again, to compare overall loss of activity to specific interruption of the BTB protein interaction domain, clonogenicity was determined using the small molecule BCL6 inhibitor FX1. Both LN18 and T98G cells were treated with either 25 or 40 μM for 24 hours, then the relative plating efficiency compared between treated and untreated cells. At 40 μM, BCL6 inhibition blocked proliferation completely, so that no colonies were formed (data not shown). At 25 μM there was a 50–70% reduction in colony formation across both cell lines (Fig 4C), consistent with the effect of the dominant negative protein.

The ability of cells to migrate was assessed using a scratch assay. Cells were allowed to form a confluent monolayer, the serum level reduced to inhibit proliferation, and then a wound scratched into the monolayer. In cells treated with FX1, migration of cells into the open scratch was reduced by 70% compared to vehicle-treated cells, again in both LN18 and T98G cells (Fig 4D).

Inhibitor studies are informative but limited. We attempted to create isogenic pairs of glioblastoma cell lines, by knock-out of the BCL6 locus in LN18 and U87-MG cells using nuclease-mediated genome editing (S2 Fig). Nuclease activity could not be detected in the adherent viable fractions of transfected LN-18 and U87-MG cells, either at 24 or 72 hours. When detached cells and debris were included in the analysis, low nuclease activity was detected at 24 h, for both LN18 and U87-MG. By 72 hours, evidence of edited loci was lost altogether, and no cell line with an edited locus could be established. This showed that cells with an edit to the BCL6 locus were generated but did not remain viable. This reinforced the hypothesis that BCL6 is essential for survival of glioblastoma cells.

### BCL6 could repress transcription in glioblastoma cell lines

**Fig 5:**
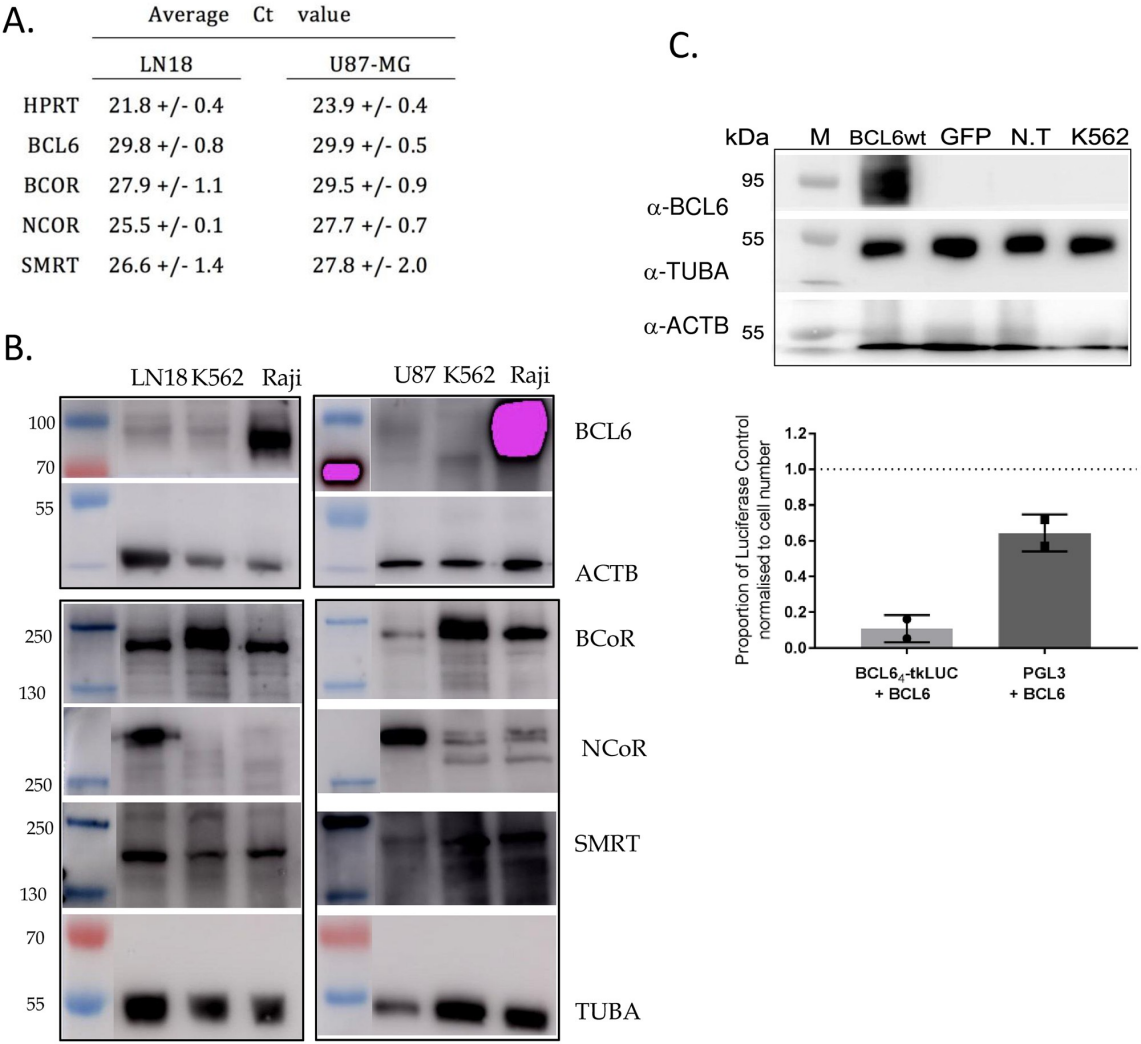
BCL6 co-repressors are expressed and functional in glioblastoma. A. Cycle thresholds for QRT-PCR for the housekeeping gene HPRT, BCL6 and co-repressors BCOR, NCOR1, and SMRT (NCOR2) in LN18 and U87-MG cells. B. Western Blot for BCOR, NCOR1, SMRT (NCOR2), alpha tubulin (TUBA), and beta actin (ACTB) in LN18, U87-MG, K562 (negative control) and Raji (positive control). **C**. Western blot for BCL6, alpha tubulin (TUBA), and beta actin (ACTB) of LN18 cells transfected with BCL6wt, GFP or non-transfected (N.T) with K562 and Raji cells as BCL6 negative and positive controls, respectively (left panel). Cells were harvested 48 hours after transfection. Luciferase assay of LN18 cells co-transfected with both BCL6_4_-tkLUC and BCL6wt plasmids or PGL3 and BCL6wt plasmids and harvested 48 hours after transfection (right panel). The luciferase values were first normalized to cell number and then expressed as a proportion of their single transfected control (either BCL6_4_-tkLUC or PGL3 alone).

BCL6 is best characterized as a transcriptional repressor, and it complexes with the co-repressors BCOR, NCOR1 and/or NCOR2 to repress transcription, via the BTB/POZ domain. The expression of the co-repressors in glioblastoma cell lines was examined by Q-RT-PCR and Western blot. All three co-repressors were expressed in all of the glioblastoma cell lines used in this study (Fig 5A and 5B), suggesting that glioblastoma cells have the necessary machinery intact for BCL6 to repress transcription. Next, functional co-repression was examined. Over-expression of BCL6 in both LN18 and U87-MG cells specifically led to the suppression of transcription driven by a BCL6 responsive element (pBCL6_4_-tk-LUC) [30] (Fig 5C), confirming that glioblastoma cells are indeed competent for transcriptional repression by BCL6.

### BCL6 was induced by the genotoxic therapies used in glioblastoma

**Fig 6:**
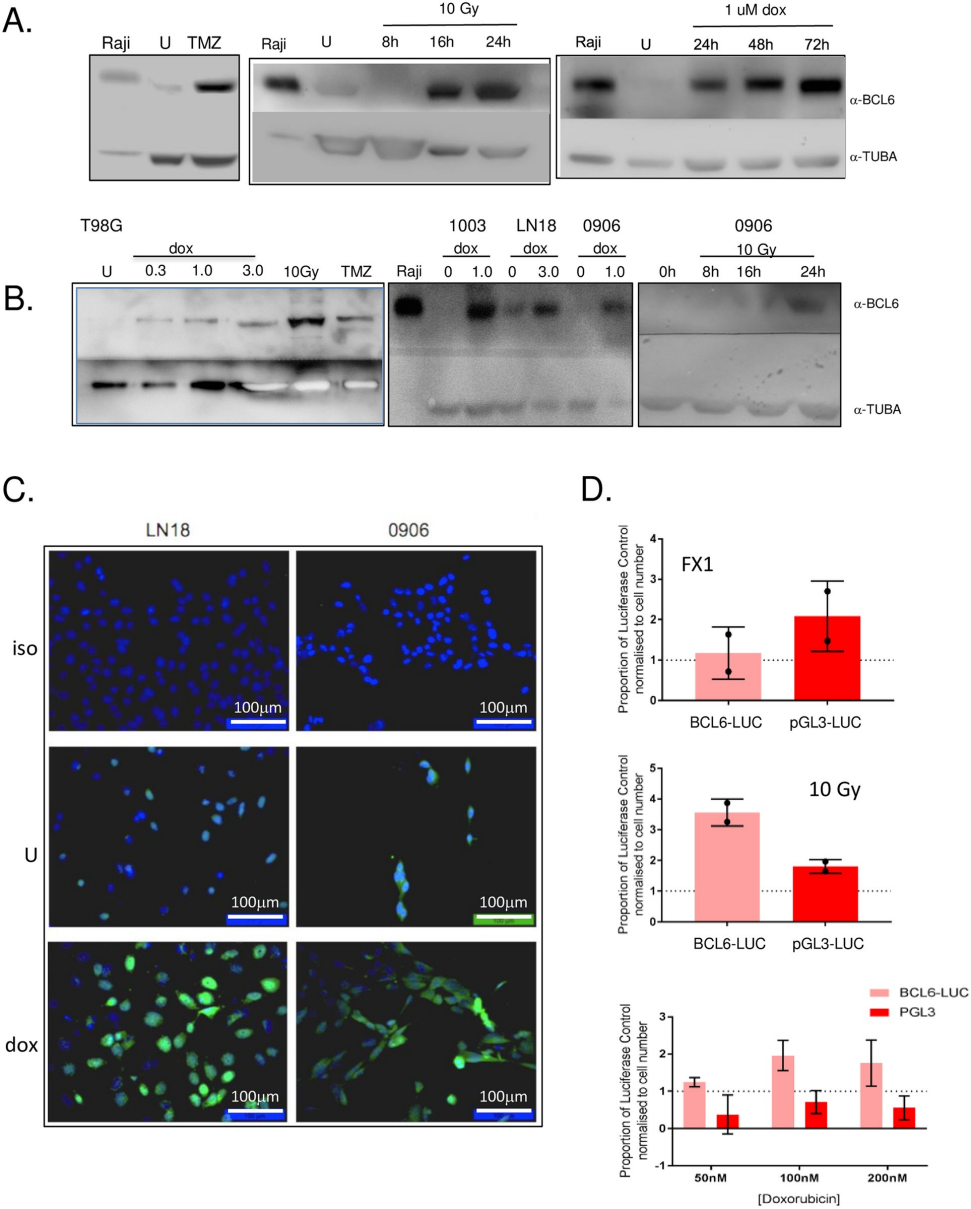
BCL6 expression was induced by DNA-damaging therapy in glioblastoma. **A**. LN18 cells were untreated (U), or treated with 10 μM temozolomide every 2 days for 12 days (7 treatments total, upper left panel), 10 Gy of ionizing radiation (upper right) or increasing doses of doxorubicin as described (lower left). LN18 cells were treated with 1 μM doxorubicin for 24–72 hours (lower right panel). BCL6 and alpha tubulin (TUBA) were detected by western blot 24 h after treatment, with Raji cell lysate used as a positive control for BCL6 expression. **B**. Cell line T98G was treated with increasing doses of doxorubicin, or 10 Gy ionizing radiation for 24 h, and with 10μM TMZ every 2 days for 12 days (left panel.) NZG-1003, LN18 and NZG-0906 were treated with doxorubicin for 24 hours (center panel), NZG-0906 was treated with 10 Gy ionizing radiation at 8, 16, 24 hours (right panel). BCL6 and alpha-tubulin were detected by western, and Raji used as a positive control. U, untreated cells. **C**. LN18 (left) and NZG-0906 (right) were grown on coverslips, left untreated (center panels) or treated with 3 μM (LN18) or 1 μM (NZG-0906) doxorubicin (lower panels), and BCL6 detected by immunofluorescence. An isotype antibody was used as a control on treated cells (upper panels). Nuclei were imaged with DAPI. All data shown are representative of at least 3 independent experiments. Cropped gel images retain all bands, immunofluorescence images were adjusted only for brightness and contrast, and were adjusted evenly over the entire image. **D**. Luciferase assay of LN18 cells transfected with either BCL64-tkLUC (light) or PGL3 (dark) plasmids and harvested 48 hours after transfection. Luciferase values were normalized to cell number then expressed as a proportion of their transfected untreated control (either BCL64-tkLUC or PGL3 alone). Cells were treated with 25 μM FX1 (top), 10 Gy ionizing radiation (middle) or doxorubicin as described (bottom). All assays were performed 48 hours after treatment.

During germinal center development, BCL6 is up-regulated in response to double-strand DNA breaks. We assessed whether DNA damaging therapy, which also induces double strand breaks, can similarly induce BCL6. The glioblastoma cell lines were treated with temozolomide, ionizing radiation and doxorubicin, then BCL6 protein level and sub-cellular localization assessed by both western blot and immunofluorescence microscopy. Consistent with Fig 1D, LN18 cells had a low but generally detectable basal level of BCL6 expression (Fig 6A). Cells were treated every 48 hours with a physiologically achievable concentration of temozolomide (10 μM), a total of 7 times over 12 days. This led to significant and substantial BCL6 induction in all cells tested—LN18 (Fig 6A), T98G, NZG-1003 and NZG-0906 (Fig 6B). Similarly, cells were exposed to 5 daily fractions of 2 Gy ionizing radiation, or one dose of 10 Gy. Both of these induced BCL6 expression in all lines tested—LN18, T98G, NZG-0906, and NZG-1003 (Fig 6). The effect of doxorubicin was also examined, because although it does not cross the blood-brain barrier *in vivo*, doxorubicin has demonstrable efficacy against glioblastoma *in vitro* [19]. All cell lines tested up-regulated BCL6 with doxorubicin exposure, similar to temozolomide and ionizing radiation. At very low dose, down to 15 nM, doxorubicin had little direct impact on viability of glioblastoma cell lines, but led to significant induction of BCL6 over 24 hours. The doxorubicin effect on BCL6 level increased with dose and was sustained at least up to 72 hours.

Immunofluorescence of doxorubicin -treated LN18 and NZG-0906 cells confirmed that induced BCL6 was predominantly nuclear, (Fig 6C), suggesting that it should be transcriptionally active. To determine whether DNA damage-induced BCL6 was also a transcriptional repressor, we repeated the BCL6-responsive luciferase reporter assay, but without transfection of wild-type BCL6. First, endogenous BCL6 was inhibited with FX1. Surprisingly, this had no effect on BCL6 reporter activity, and in fact led to less expression from the BCL6 responsive reporter than from the control reporter. If BCL6 repressed transcription, inhibition should have released this and increased luciferase expression. To look at this further, reporter-transfected cells were treated with 10 Gy of ionizing radiation. This caused a robust increase in BCL6 transcriptional activity not seen on the control reporter, which suggested that induced GBM BCL6 might increase transcription. This was confirmed in cells treated with doxorubicin, which also had increased BCL6 reporter activity. Together, these data suggest that BCL6 induced by therapy in glioblastoma is transcriptionally active, but may not have the classic ‘transcriptional repressor’ activity observed in B cells.

**Fig 7:**
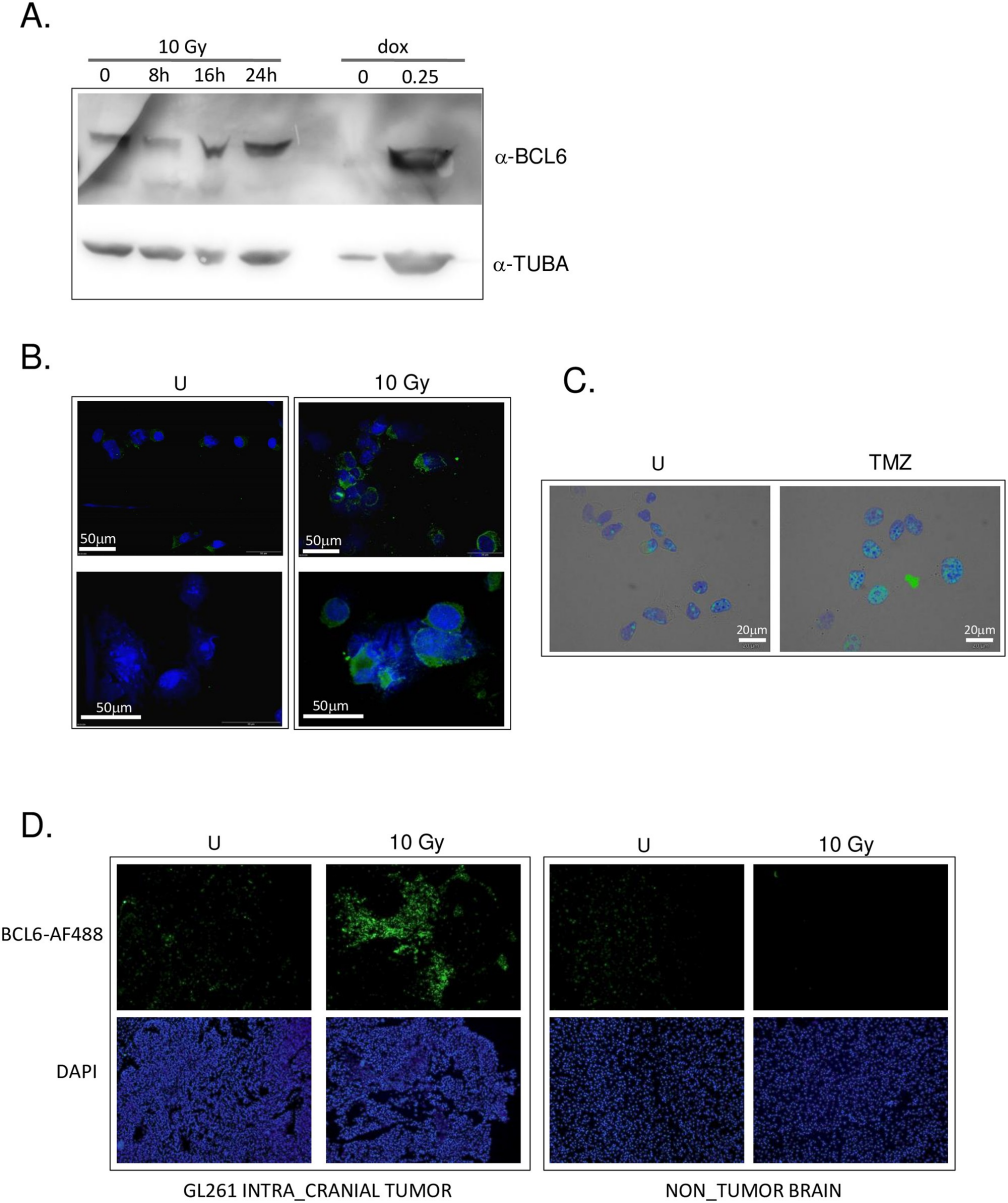
BCL6 was up-regulated by DNA damaging therapy in the transplantable murine glioma model GL261. **A**. GL261 cells were treated with 10 Gy of ionizing radiation or 0.25 μM doxorubicin, and harvested at the time point shown (10 Gy) or at 24 hours post-treatment (dox). After electrophoresis and transfer, membranes were cut and blotted with anti-BCL6 (top) or anti-alpha tubulin (bottom). Image representative of at least 3 independent experiments. **B**. GL261 cells grown on coverslips were treated with 10 Gy of ionizing radiation, then fixed and stained with DAPI (blue) and anti-BCL6 antibody (green). Immunofluorescence images were adjusted only for brightness and contrast, and were adjusted evenly over the entire image. All images were adjusted exactly the same way, and all images representative of at least 3 independent experiments. **C**. GL261 cells on coverslips were treated with 7 doses of 10 μM TMZ on alternate days, then fixed and stained with DAPI (blue) and anti-BCL6 antibody (green). Immunofluorescence images were adjusted only for brightness and contrast, and were adjusted evenly over the entire image. All images were adjusted exactly the same way, and all images representative of at least 3 independent experiments **D**. Brains from mice bearing intra-cranial GL261 tumors prior to (U), and 48 hours post irradiation (10 Gy) were collected and BCL6 expression in the tumor and the normal brain determined by immunofluorescence microscopy using Alexa-488-labelled anti-BCL6 antibody. Nuclei were imaged with DAPI. All data shown are representative of at least 3 independent experiments. Cropped gel images retain all bands, immunofluorescence images were adjusted only for brightness and contrast, and were adjusted evenly over the entire image.

The effect of therapy on BCL6 expression was assessed *in vivo* with an orthotopic murine model using the GL261 cell line. Notably, GL261 cells were much more sensitive to DNA damaging therapy than the human cell lines—10x less doxorubicin (0.25 μM rather than 1–3 μM) was required to injure these cells, and the standard radiation and TMZ regimens left many fewer surviving cells. Despite this, BCL6 protein was increased in those GL261 cells that survived doxorubicin, radiation and temozolomide treatment *in vitro* (Fig 7A). Unlike the human cells, the sub-cellular localization of BCL6 in GL261 was largely cytoplasmic, both before and after DNA damage induced by therapy (Fig 7B and 7C).

GL261 cells were implanted intra-cranially into one hemisphere of C57/BL6 mice. Once significant tumor burden was demonstrated by the onset of weight loss, 10 Gy of whole brain irradiation was delivered as described previously [31] and brains collected 24 or 48 hours after irradiation. Localization of tumor within the brain was determined by DAPI-staining of nuclei, which showed the densely packed irregular nuclei typical of this model [19]. BCL6 protein was analyzed by immunofluorescence in the brain tumor, and compared to normal brain in the opposite hemisphere (Fig 7D). There was a low level of basal BCL6 expression in the tumors, which was very prominent by 48 hours. BCL6 was not observed in the normal brain tissue, either before or after irradiation.

### BCL6 blockade with genotoxic therapies enhanced their efficacy

**Fig 8:**
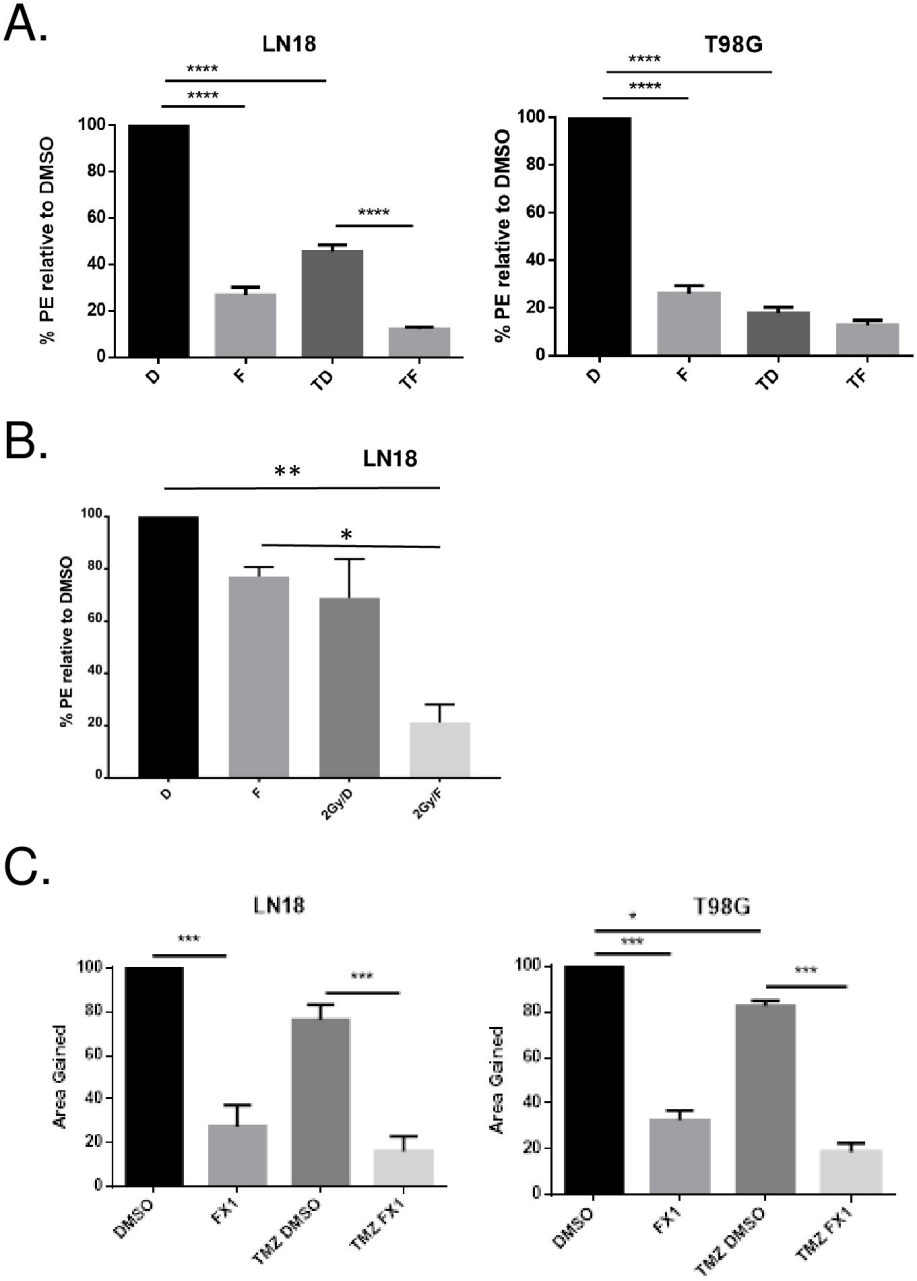
BCL6 inhibition increased the efficacy of chemotherapy and radiation. **A**. Long-term proliferative potential was measured by clonogenic assay for LN18 (left) and T98G (right) cells with vehicle control (DMSO, D), 25 μM FX1 (F), 400 μM temozolomide with DMSO (TD), and temozolomide plus 25 μM FX1 (TF). Plating efficiency (PE) was normalized to the DMSO treated cells, and data are averaged from three independent replicate experiments. Error bar represents SEM, asterisks indicate level of statistical significance by ANOVA. **B**. Clonogenic assay for LN18 cells treated with 2 Gy ionizing radiation, as described above. **C**. Cell migration was measured by scratch healing assay in LN18 (left) and T98G (right) cells treated with vehicle control (DMSO), 25 μM FX1 (FX1), 400 μM temozolomide with vehicle (TMZ DMSO), and 400 μM temozolomide plus FX1 (TMZ FX1). Data are averaged from three independent replicates and normalized to the effect of the vehicle DMSO. Error bar represents SEM, asterisks indicate level of statistical significance by ANOVA.

The induction of BCL6 by genotoxic chemotherapy and radiation implied that BCL6 might turn off cell death pathways such as apoptosis, that should be activated by high levels of DNA damage. If this was the case, concomitant BCL6 inhibition should increase the efficacy and toxicity of DNA damaging therapies. To test this, LN18 and T98G cells were exposed to either temozolomide or ionizing radiation, with and without the small molecule BCL6 inhibitor FX1. Clonogenicity assays showed that the combination of FX1 and temozolomide had an additive, or even greater-than-additive, effect on the temozolomide suppression of colony formation, both in LN18 and in T98G cells (Fig 8A). Similarly, BCL6 inhibition added to ionizing radiation had a greater effect than radiation alone in LN18 cells (Fig 8B).

The effect of BCL6 inhibition on cellular migration was also determined (Fig 8C). BCL6 inhibition with FX1 significantly reduced the ability of both LN18 and T98G cells to migrate into a ‘scratch’ wound in a the cell monolayer. Temozolomide by itself had little effect on migration, but as in the clonogenicity data, combining BCL6 inhibition with temozolomide reduced migration further, in both cell types. These data are consistent with our hypothesis that BCL6 up-regulation in response to DNA damaging therapy contributes to the resistance of glioblastoma cells to that therapy.

## Discussion

In this study, we assessed the spectrum of BCL6 protein in glioblastoma survival and therapy resistance, and demonstrated a key role for BCL6 in survival of glioblastoma cells *in vitro* after DNA damage. We found cells with endogenous BCL6 present in every glioblastoma tumor examined, although in some sections, the percentage of positive cells was very low. The localization of BCL6 in glioblastoma was particularly interesting—in many cases, it appeared to localize to perivascular regions of glioblastoma. If this is confirmed upon further analysis it might suggest BCL6 is enriched in cancer stem cells that inhabit the perivascular niche [32]. Co-staining BCL6 with GBM cancer stem cell markers such as CD133, integrin a6, notch or IL8 receptors [33, 34] would support this.

A previous observation linking glioma and BCL6 [12] saw a correlation between BCL6 expression and p53, predominantly in BCL6-translocated tumors. We did not see any correlation between p53 status and BCL6 expression. This may be related to our tumor cohort—p53 mutation is much more common in secondary glioblastoma, and our tumors were predominantly primary disease. Similarly there was also no overt relationship between BCL6 level and IDH1 mutation, another mutation more common in secondary glioblastoma. Less than 10% of our tumors had any mutation in IDH1 or IDH2, and all 5 were IDH1 R132H. An interesting correlation was observed between BCL6 and MGMT expression, with higher BCL6 expression observed in MGMT positive tumors. Recent data show MGMT is commonly expressed by macrophages, and some tumors classified as MGMT-positive actually reflect high levels of MGMT+ macrophage infiltration [35]. The observed association between MGMT and BCL6 could be due to BCL6 expression in the infiltrating macrophages. However, we examined this carefully and are confident that BCL6 expression reported here is in the tumor cells, and not any immune compartment. Further, retention of expression of BCL6 in cell lines underlies the importance of BCL6 in tumor cells. The nature of the relationship between BCL6 and MGMT expression in glioblastoma is unclear, but could provide a double hit of therapy resistance—MGMT would repair the TMZ-mediated damage, while BCL6 induced by radiotherapy and TMZ would enhance survival. This becomes particularly critical if the cells expressing BCL6 are cancer stem cells.

In line with the enrichment of the apoptosis gene set in the differentially expressed genes, and published data, we saw a highly reproducible increase in apoptosis that did not depend on the method of BCL6 inhibition—siRNA, inhibitors and dominant negative mutants all increased apoptosis. However, the number of apoptotic cells was small, indicating that while BCL6 has some role in preventing apoptosis, supported by previous observations [12], it is not entirely responsible for poor apoptosis in glioblastoma. However, apoptosis is not essential in cancer therapy, and perhaps not even desirable—apoptotic cell death tends to be immunologically silent [36], which may prevent naturally occurring anti-tumor immunity from contributing to the therapeutic response. We took an agnostic approach to cell death and measured just acute loss of viability, and long-term proliferative potential. Again using multiple modes of inhibition, we clearly demonstrated that BCL6 is very important in glioblastoma cell survival—BCL6 inhibition using the small molecule FX1 at 40 μM stopped all long-term proliferation, and even at 25 μM there was significant suppression of colony formation. Further, cell viability was lost completely when the BCL6 locus was knocked out. These findings were consistent with the published reports [13], although contrary to Xu *et al*. we never saw evidence of senescence in any of our BCL6-inhibited cells. Our gene expression data also suggest that there are other pro-survival pathways, such as MTORC1 signaling, that are lost when endogenous BCL6 activity is blocked. These could also contribute to the protective effect of BCL6 in glioblastoma. Even in the cell lines that had low basal levels of endogenous BCL6, DNA damaging therapy induced substantial increases in BCL6. This appears to be a conserved response to stress [37] acquired by glioblastoma that drives extensive resistance to therapy.

Another aspect of therapy resistance, and also intriguingly of the cancer stem cell phenotype, is migration and invasion. In glioblastoma, cells that have migrated beyond the main body of the tumour cannot be surgically resected, and those that have moved beyond the radiation field will escape DNA damage. These data demonstrated that endogenous BCL6 also drives the highly migratory phenotype of glioblastoma cells, emphasizing the importance of BCL6 as a target for therapy.

Our data are largely *in vitro*. Our *in vivo* model has limitations that made further analysis of BCL6 unfeasible. BCL6 was robustly up-regulated in response to DNA damage, but that was where the similarity to the human cell lines ended. First, BCL6 in GL261 was predominantly cytoplasmic even after DNA damaging therapy, unlike the other cell lines. This made it unlikely to directly regulate transcription. Secondly, the GL261 cells were much more sensitive to DNA damaging therapies, with 10 times less doxorubicin required for the same level of cytotoxicity, and more cell death observed with ionizing radiation. Combined, these data implied that BCL6 survival activity is not conserved in the GL261 cells. Thus the animal model, which we and others have used widely [19, 31, 38–40], would not be useful, beyond demonstrating that DNA damaging therapy did indeed induce BCL6 *in vivo* as well as *in vitro*.

In order to understand whether therapy-induced BCL6 is a transcriptional repressor in glioblastoma, we used the classic BCL6 responsive luciferase reporter system [41]. This clearly demonstrated that while over-expressed BCL6 can repress transcription, therapy-induced BCL6 might increase transcription from BCL6 binding sites. This was surprising but is supported by the literature. Although not directly highlighted by the authors, activation of gene expression by BCL6 was observed by Xu *et al* [13]. In addition PLZF, a BTB-POZ domain protein closely related to BCL6, acts as either repressor or activator, in different cell types. This is dictated by post-translational modification—acetylation and phosphorylation of key amino acids [42]. Whether BCL6 similarly directly activates transcription in glioblastoma has not yet been determined.

## Conclusions

The data presented here strongly support the hypothesis that BCL6 drives survival in glioblastoma. Importantly, we demonstrated that BCL6 is induced by the therapies used to treat glioblastoma, and that the pro-survival activity of ‘therapy-induced’ BCL6 helps drive the intrinsic resistance to therapy observed in this disease. People diagnosed with glioblastoma have few options for treatment, and most of those options are ineffective at best. BCL6 inhibition has been shown to be highly effective in animal models of lymphoma and leukemia, and several inhibitors are currently in clinical development. BCL6 inhibition in glioblastoma is highly feasible—several inhibitors, both peptide and small molecule, have been developed for BCL6 [4, 28], including some that should pass through the blood-brain barrier and accumulate in the brain. We believe that addition of BCL6 inhibition to the standard protocols of chemo-radiation has real potential to improve the outcome for people with glioblastoma.

## Supporting information

S1 Fig Basal BCL6 localization in primary GBM cell lines. Blue = DAPI, Green = BCL6. Brightness and contrast was adjusted on merged images. The same adjustments were made to the whole image and to each image the same way.
(TIF)

S2 Fig ZFN editing of BCL6 locus. Heteroduplex formation of PCR products Spanning the BCL6 ZFN site. The surveyor kit was used to identify presence of loci with deletions induced by ZFn against the BCL6 locus. Brightness and contrast of the original gel image (lower left corner) was altered to visualize the short products (asterisks) resulting in cleavage of heteroduplex by the Surveyor enzyme. Non-transfected (No ZFN) or tranfected cells (Adherent only, or adherent and floating) were analysed at the indicated time-points post-transfection, as descrided.
(TIF)

S3 Fig (PDF)

S4 Fig Original western blots from Fig 6. The upper part of each membrane was incubated with 1:500 (3% skim milk) of anti-BCI6 monoclonal antibody D8 and lower part 1:2,0000 (3% skim milk) of anti-tubulin. Goat anti-moise IgG HRP secondary antibody was used at 1:10,000 (3% skim milk). Detection by enhanced chemiluminescence (Ultrasignal ECL kit, pierce), imaged using the Gel logic 4000 PRO Imaging System (Carestream, Rochester, NY USA).
(TIF)

S5 Fig Original western blots from Fig 7. The upper part of each membrane was incubated with 1:500 (3% skim milk) of anti-BCI6 monoclonal antibody D8 and lower part 1:2,0000 (3% skim milk) of anti-tubulin. Goat anti-moise IgG HRP secondary antibody was used at 1:10,000 (3% skim milk). Detection by enhanced chemiluminescence (Ultrasignal ECL kit, pierce), imaged using the Gel logic 4000 PRO Imaging System (Carestream, Rochester, NY USA).
(TIF)
