# Supplementary figures and images for: The oncogene BCL6 is up-regulated in glioblastoma in response to DNA damage, and drives survival after therapy
Source: PLoS One. 2020 Apr 22;15(4):e0231470. doi: 10.1371/journal.pone.0231470 (PMC7176076; doi:10.1371/journal.pone.0231470)

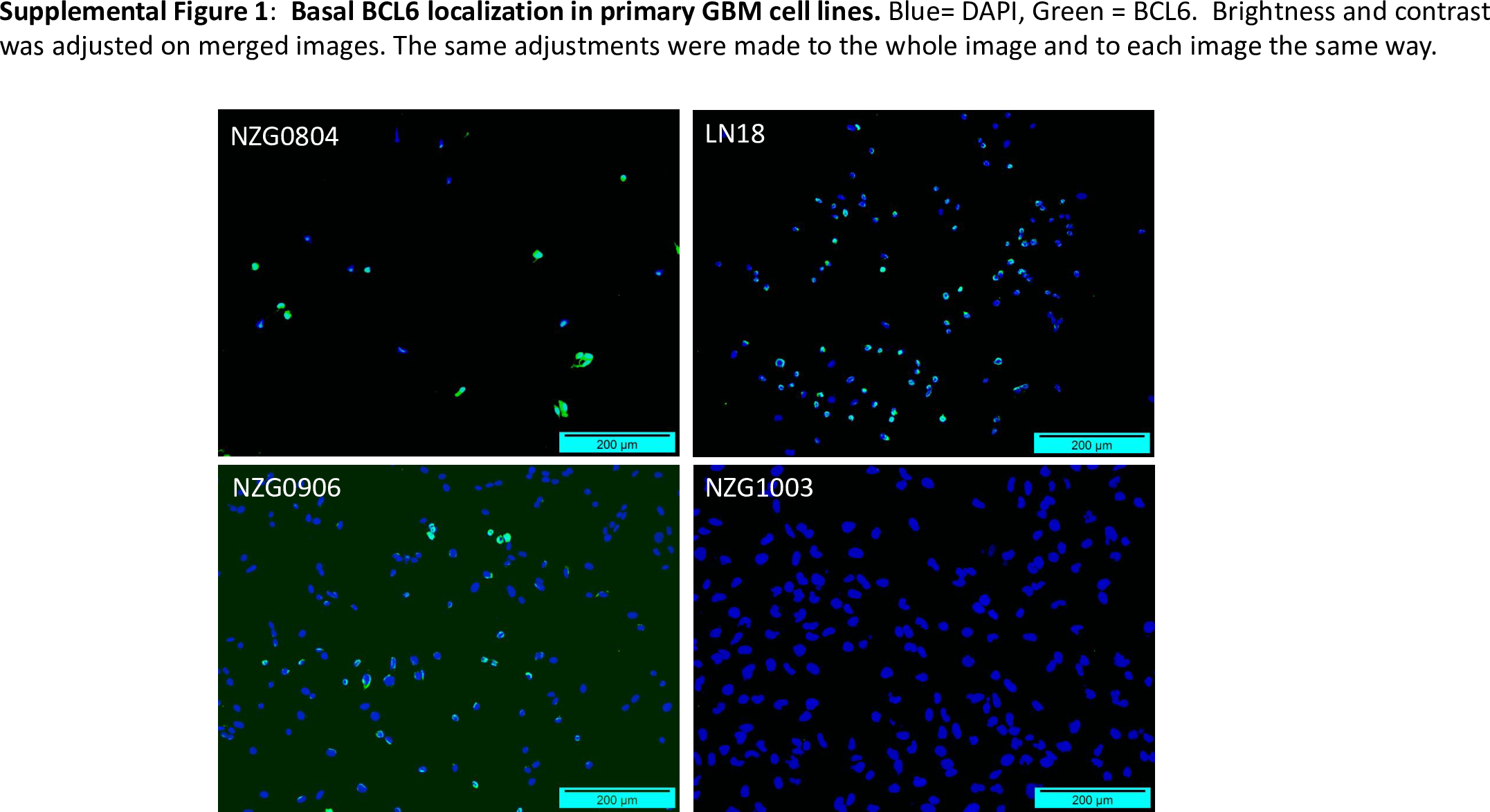

Supplement: S1 Fig — Blue = DAPI, Green = BCL6. Brightness and contrast was adjusted on merged images. The same adjustments were made to the whole image and to each image the same way. (TIF) [file pone.0231470.s001.tif]

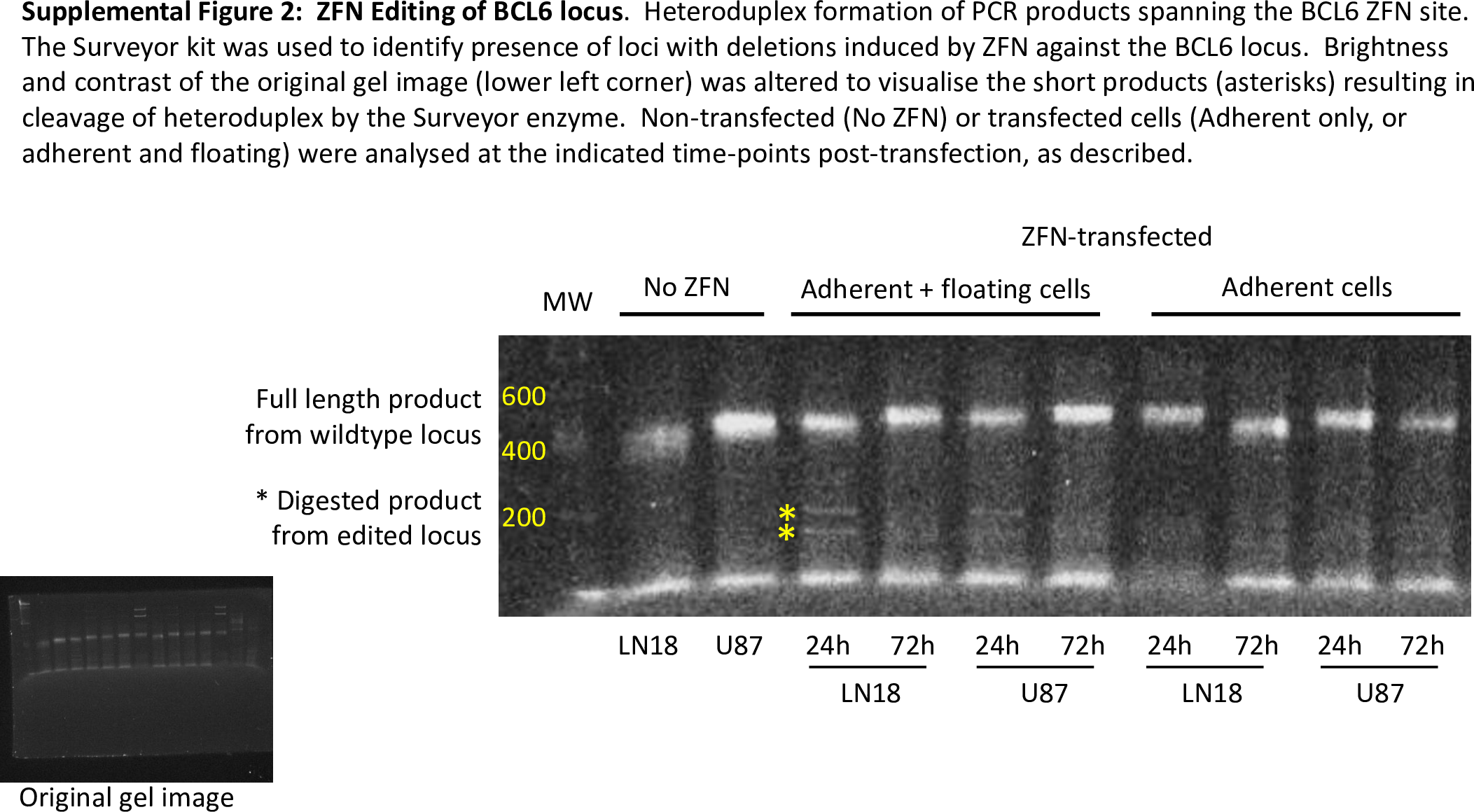

Supplement: S2 Fig — Heteroduplex formation of PCR products Spanning the BCL6 ZFN site. The surveyor kit was used to identify presence of loci with deletions induced by ZFn against the BCL6 locus. Brightness and contrast of the original gel image (lower left corner) was altered to visualize the short products (asterisks) resulting in cleavage of heteroduplex by the Surveyor enzyme. Non-transfected (No ZFN) or tranfected cells (Adherent only, or adherent and floating) were analysed at the indicated time-points post-transfection, as descrided. (TIF) [file pone.0231470.s002.tif]

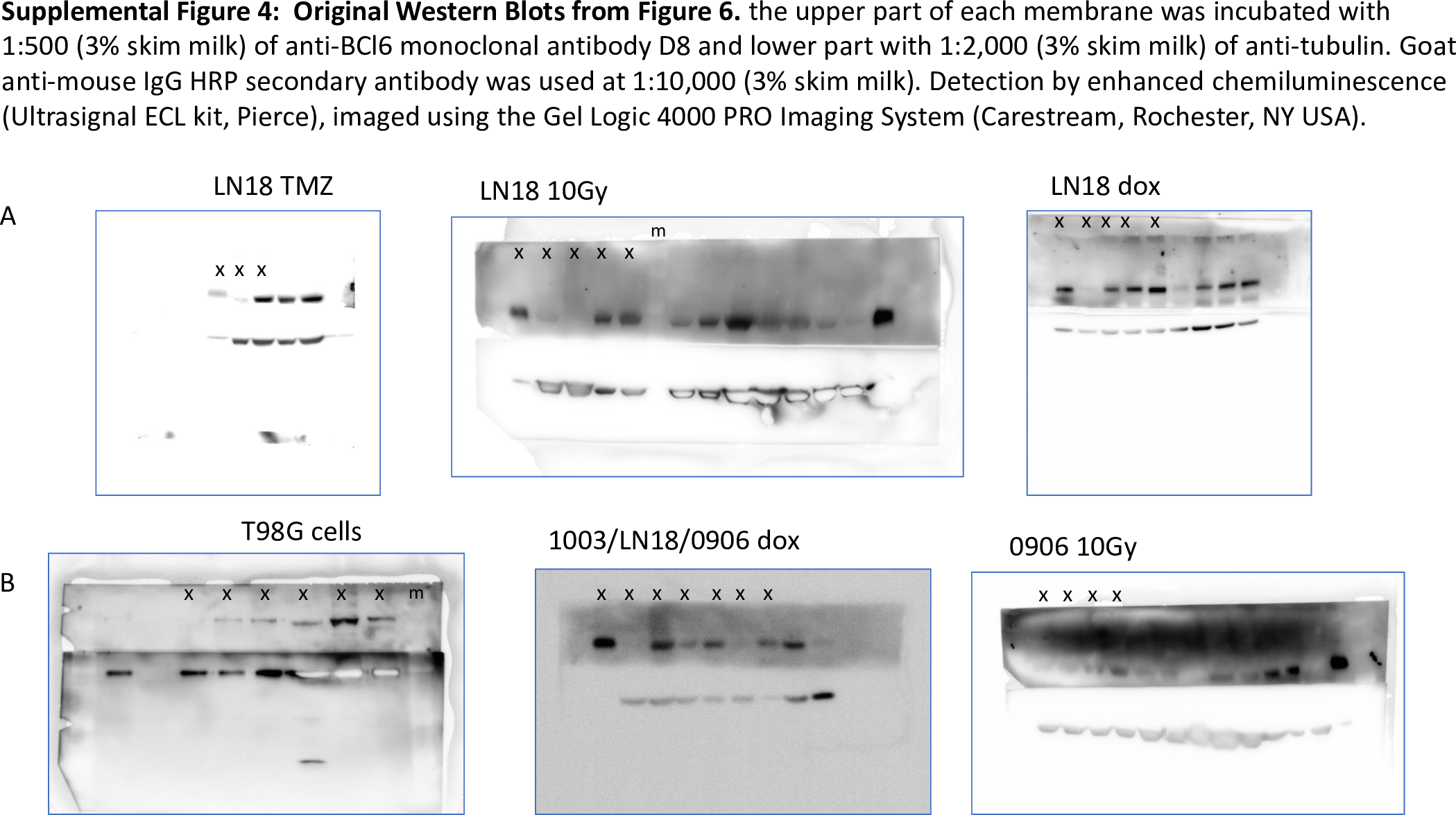

Supplement: S4 Fig — The upper part of each membrane was incubated with 1:500 (3% skim milk) of anti-BCI6 monoclonal antibody D8 and lower part 1:2,0000 (3% skim milk) of anti-tubulin. Goat anti-moise IgG HRP secondary antibody was used at 1:10,000 (3% skim milk). Detection by enhanced chemiluminescence (Ultrasignal ECL kit, pierce), imaged using the Gel logic 4000 PRO Imaging System (Carestream, Rochester, NY USA). (TIF) [file pone.0231470.s004.tif]

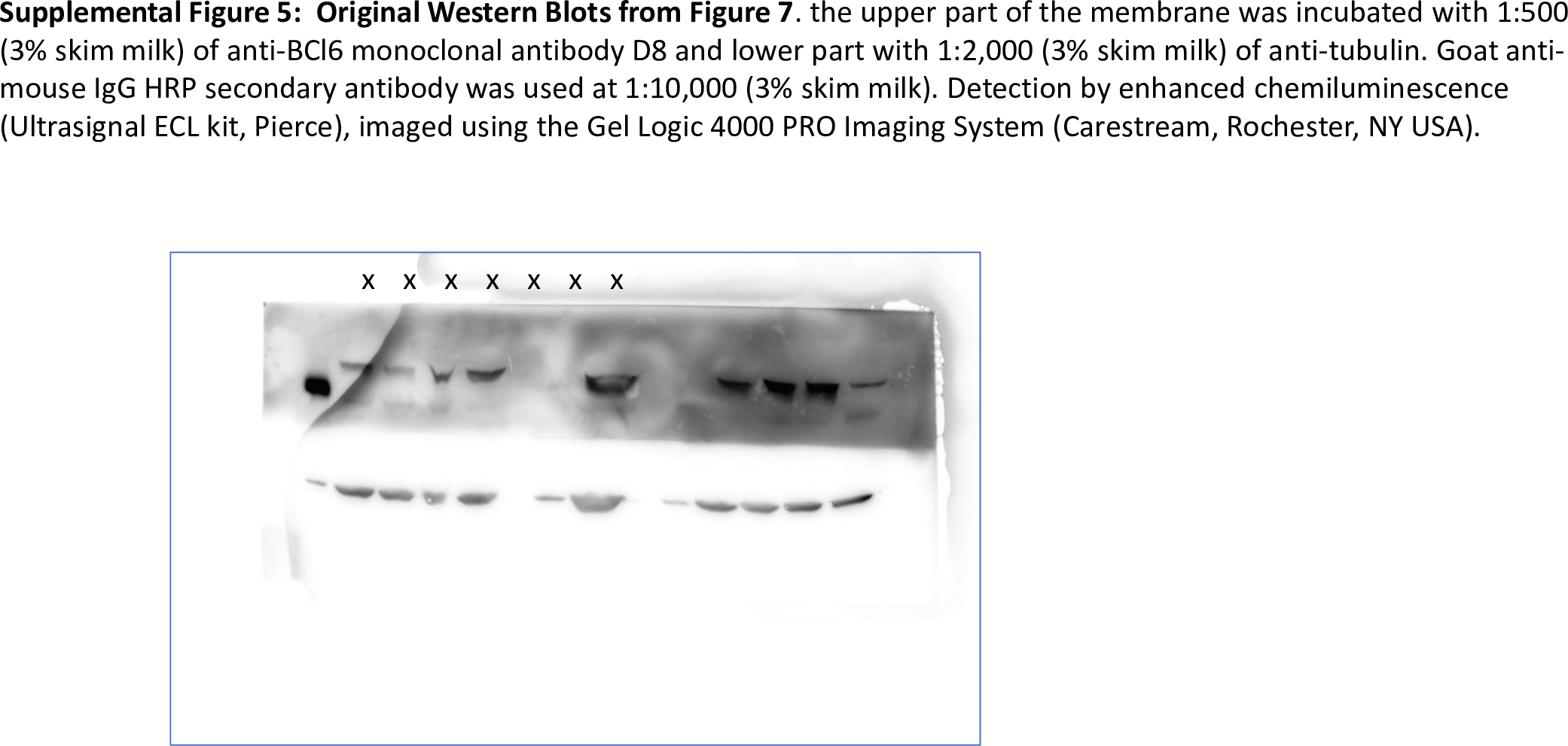

Supplement: S5 Fig — The upper part of each membrane was incubated with 1:500 (3% skim milk) of anti-BCI6 monoclonal antibody D8 and lower part 1:2,0000 (3% skim milk) of anti-tubulin. Goat anti-moise IgG HRP secondary antibody was used at 1:10,000 (3% skim milk). Detection by enhanced chemiluminescence (Ultrasignal ECL kit, pierce), imaged using the Gel logic 4000 PRO Imaging System (Carestream, Rochester, NY USA). (TIF) [file pone.0231470.s005.tif]
