# Supplementary material for: The oncogene BCL6 is up-regulated in glioblastoma in response to DNA damage, and drives survival after therapy
Source: PLoS One. 2020 Apr 22;15(4):e0231470. doi: 10.1371/journal.pone.0231470 (PMC7176076; doi:10.1371/journal.pone.0231470)
Supplement: S3 Fig — (PDF) [file pone.0231470.s003.pdf]

Figure 5B: Blot BCL6, LN18 cells

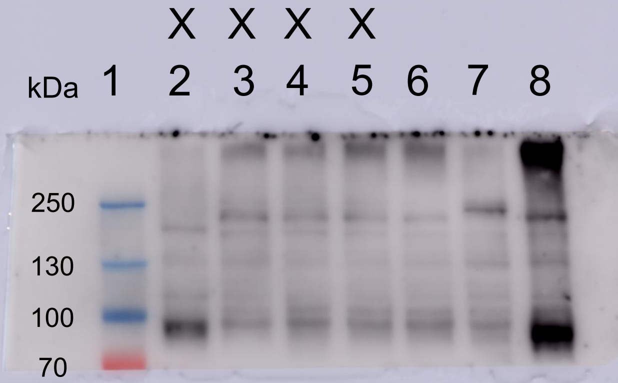

1: PageRuler plus prestained ladder
2: 3 uM Doxorubicin (LN18 cells)
3: 10 Gy Irradiation (LN18 cells)
4: 25 uM FX1 (LN18 cells)
5: DMSO (equiv. volume to FX1, LN18 cells)
6: Untreated LN18
7: Untreated K562 cells
8: Untreated Raji cells
Imaged using Western Lightning Ultra ECL kit, on Amersham Imager 600

Figure 5B: Blot ACTB, LN18 cells

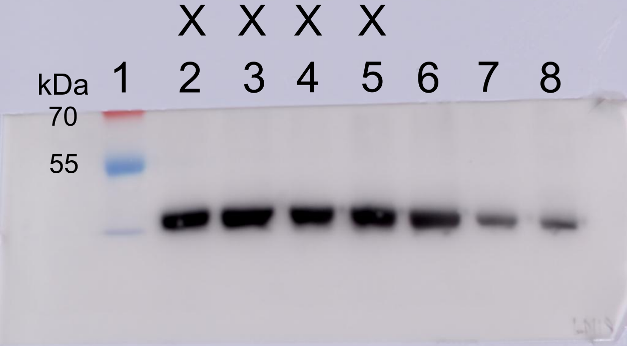

1: PageRuler plus prestained ladder
2: 3 uM Doxorubicin (LN18 cells)
3: 10 Gy Irradiation (LN18 cells)
4: 25 uM FX1 (LN18 cells)
5: DMSO (equiv. volume to FX1, LN18 cells)
6: Untreated LN18
7: Untreated K562 cells
8: Untreated Raji cells

Imaged using Western Lightning Ultra ECL kit, on Amersham Imager 600

Figure 5B: Blot BCL6, U87 cells

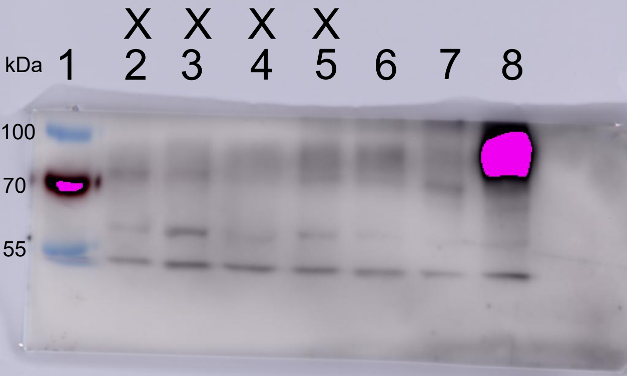

1: PageRuler plus prestained ladder
2: 3 uM Doxorubicin (U87 cells)
3: 10 Gy Irradiation (U87 cells)
4: 25 uM FX1 (U87 cells)
5: DMSO (equiv. vol. to FX1, U87 cells)
6: Untreated U87 cells
7: Untreated K562 cells
8: Untreated Raji cells
Imaged using Western Lightning Ultra ECL kit, on Amersham Imager 600

Figure 5B: Blot ACTB, U87 cells

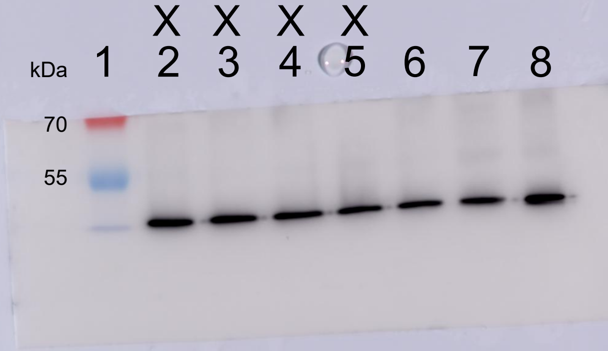

1: PageRuler plus prestained ladder
2: 3 uM Doxorubicin (U87 cells)
3: 10 Gy Irradiation (U87 cells)
4: 25 uM FX1 (U87 cells)
5: DMSO (equiv. vol. to FX1, U87 cells)
6: Untreated U87 cells
7: Untreated K562 cells
8: Untreated Raji cells

Imaged using Western Lightning Ultra ECL kit, on Amersham Imager 600

Figure 5B: Blot BCOR, LN18 cells

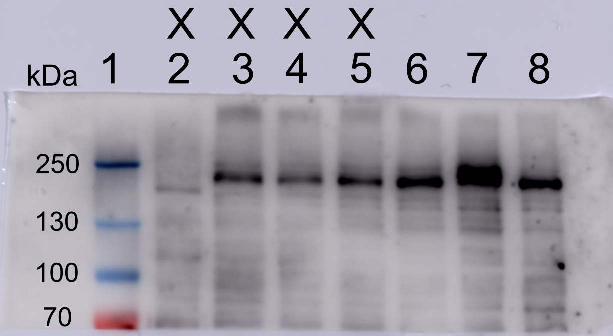

1: PageRuler plus prestained ladder
2: 3 uM Doxorubicin (LN18 cells)
3: 10 Gy Irradiation (LN18 cells)
4: 25 uM FX1 (LN18 cells)
5: DMSO (equiv. volume to FX1, LN18 cells)
6: Untreated LN18
7: Untreated K562 cells
8: Untreated Raji cells
Imaged using Western Lightning Ultra ECL kit, on Amersham Imager 600

Figure 5B: Blot NCOR, LN18 cells

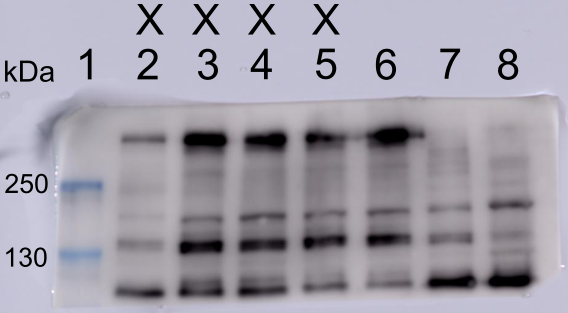

1: PageRuler plus prestained ladder
2: 3 uM Doxorubicin (LN18 cells)
3: 10 Gy Irradiation (LN18 cells)
4: 25 uM FX1 (LN18 cells)
5: DMSO (equiv. volume to FX1, LN18 cells)
6: Untreated LN18
7: Untreated K562 cells
8: Untreated Raji cells
Imaged using Western Lightning Ultra ECL kit, on Amersham Imager 600

Figure 5B: Blot SMRT, LN18 cells

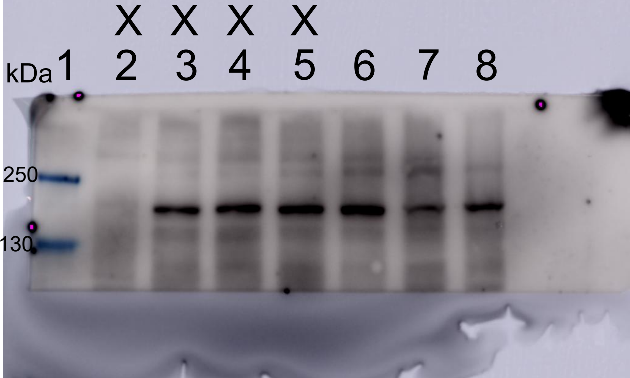

1: PageRuler plus prestained ladder
2: 3 uM Doxorubicin (LN18 cells)
3: 10 Gy Irradiation (LN18 cells)
4: 25 uM FX1 (LN18 cells)
5: DMSO (equiv. volume to FX1, LN18 cells)
6: Untreated LN18
7: Untreated K562 cells
8: Untreated Raji cells
Imaged using Western Lightning Ultra ECL kit, on Amersham Imager 600

Figure 5B: Blot TUBA, LN18 cells

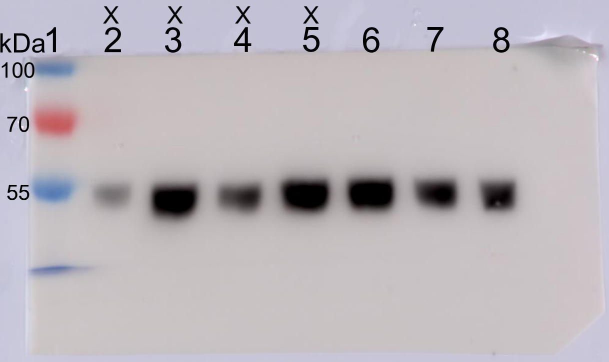

1: PageRuler plus prestained ladder
2: 3 uM Doxorubicin (LN18 cells)
3: 10 Gy Irradiation (LN18 cells)
4: 25 uM FX1 (LN18 cells)
5: DMSO (equiv. volume to FX1, LN18 cells)
6: Untreated LN18
7: Untreated K562 cells
8: Untreated Raji cells

Imaged using Western Lightning Ultra ECL kit, on Amersham Imager 600

Figure 5B: Blot BCOR, U87 cells

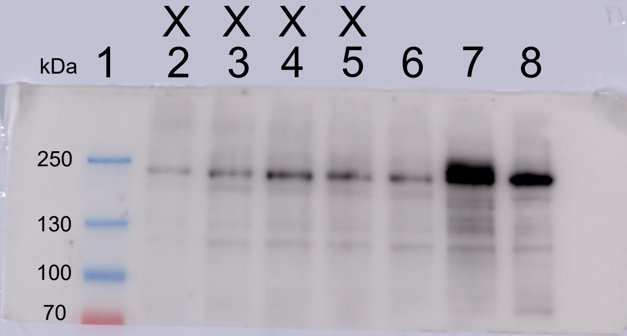

1: PageRuler plus prestained ladder
2: 3 uM Doxorubicin (U87 cells)
3: 10 Gy Irradiation (U87 cells)
4: 25 uM FX1 (U87 cells)
5: DMSO (equiv. vol. to FX1, U87 cells)
6: Untreated U87 cells
7: Untreated K562 cells
8: Untreated Raji cells

Imaged using Western Lightning Ultra ECL kit, on Amersham Imager 600

Figure 5B: Blot NCOR, U87 cells

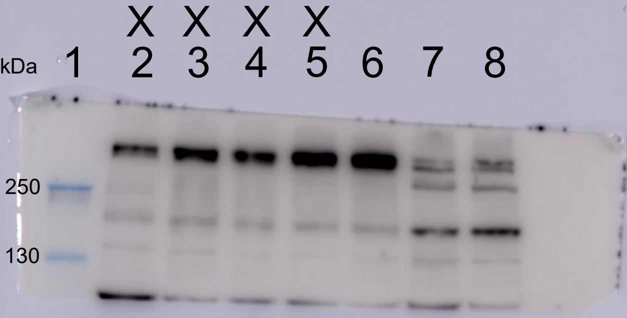

1: PageRuler plus prestained ladder
2: 3 uM Doxorubicin (U87 cells)
3: 10 Gy Irradiation (U87 cells)
4: 25 uM FX1 (U87 cells)
5: DMSO (equiv. vol. to FX1, U87 cells)
6: Untreated U87 cells
7: Untreated K562 cells
8: Untreated Raji cells

Imaged using Western Lightning Ultra ECL kit, on Amersham Imager 600

Figure 5B: Blot SMRT, U87 cells

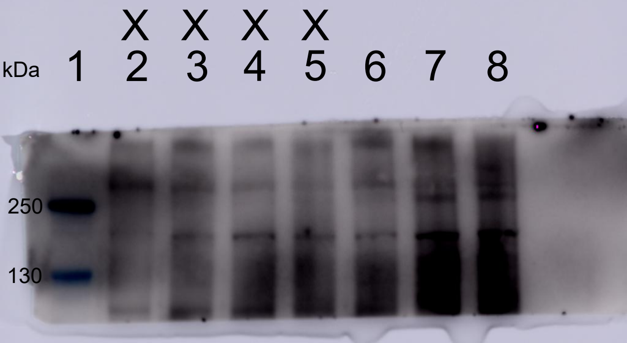

1: PageRuler plus prestained ladder
2: 3 uM Doxorubicin (U87 cells)
3: 10 Gy Irradiation (U87 cells)
4: 25 uM FX1 (U87 cells)
5: DMSO (equiv. vol. to FX1, U87 cells)
6: Untreated U87 cells
7: Untreated K562 cells
8: Untreated Raji cells

Imaged using Western Lightning Ultra ECL kit, on Amersham Imager 600

Figure 5B: Blot TUBA, U87 cells

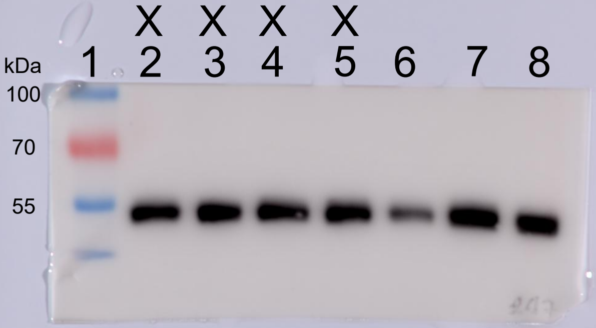

1: PageRuler plus prestained ladder
2: 3 uM Doxorubicin (U87 cells)
3: 10 Gy Irradiation (U87 cells)
4: 25 uM FX1 (U87 cells)
5: DMSO (equiv. vol. to FX1, U87 cells)
6: Untreated U87 cells
7: Untreated K562 cells
8: Untreated Raji cells

Imaged using Western Lightning Ultra ECL kit, on Amersham Imager 600

Figure 5C: Blot BCL6

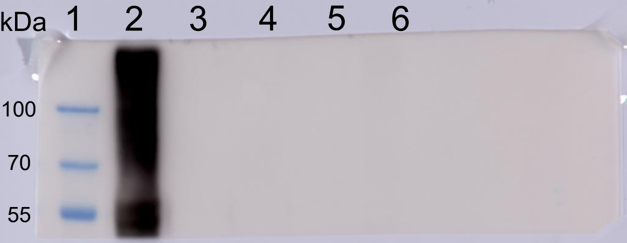

1: PageRuler plus prestained ladder
2: BCL6wt transfected LN18 cells
3: GFP transfected LN18 cells
4: Untreated LN18 cells
5: Untreated K562 cells
6: Untreated Raji cells

Imaged using Western Lightning Ultra ECL kit, on Amersham Imager 600

Figure 5C: Blot TUBA

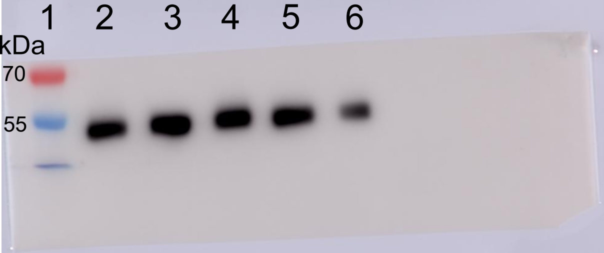

1: PageRuler plus prestained ladder
2: BCL6wt transfected LN18 cells
3: GFP transfected LN18 cells
4: Untreated LN18 cells
5: Untreated K562 cells
6: Untreated Raji cells

Imaged using Western Lightning Ultra ECL kit, on Amersham Imager 600

Figure 5C: Blot ACTB

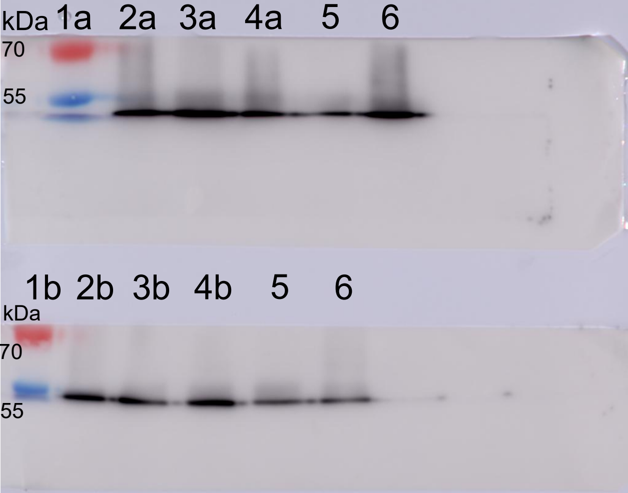

1: PageRuler plus prestained ladder. a) LN18, b) U87
2: BCL6wt transfected LN18 cells. a) LN18, b) U87
3: GFP transfected LN18 cells. a) LN18, b) U87
4: Untreated LN18 cells. a) LN18, b) U87
5: Untreated K562 cells
6: Untreated Raji cells
Imaged using Western Lightning Ultra ECL kit, on Amersham Imager 600
